# Gene Expression in a *Drosophila* Model of Mitochondrial Disease

**DOI:** 10.1371/journal.pone.0008549

**Published:** 2010-01-06

**Authors:** Daniel J. M. Fernández-Ayala, Shanjun Chen, Esko Kemppainen, Kevin M. C. O'Dell, Howard T. Jacobs

**Affiliations:** 1 Institute of Medical Technology and Tampere University Hospital, University of Tampere, Tampere, Finland; 2 Faculty of Biomedical and Life Sciences, University of Glasgow, Glasgow, United Kingdom; University of Florida, United States of America

## Abstract

**Background:**

A point mutation in the *Drosophila* gene *technical knockout* (*tko*), encoding mitoribosomal protein S12, was previously shown to cause a phenotype of respiratory chain deficiency, developmental delay, and neurological abnormalities similar to those presented in many human mitochondrial disorders, as well as defective courtship behavior.

**Methodology/Principal Findings:**

Here, we describe a transcriptome-wide analysis of gene expression in *tko^25t^* mutant flies that revealed systematic and compensatory changes in the expression of genes connected with metabolism, including up-regulation of lactate dehydrogenase and of many genes involved in the catabolism of fats and proteins, and various anaplerotic pathways. Gut-specific enzymes involved in the primary mobilization of dietary fats and proteins, as well as a number of transport functions, were also strongly up-regulated, consistent with the idea that oxidative phosphorylation OXPHOS dysfunction is perceived physiologically as a starvation for particular biomolecules. In addition, many stress-response genes were induced. Other changes may reflect a signature of developmental delay, notably a down-regulation of genes connected with reproduction, including gametogenesis, as well as courtship behavior in males; logically this represents a programmed response to a mitochondrially generated starvation signal. The underlying signalling pathway, if conserved, could influence many physiological processes in response to nutritional stress, although any such pathway involved remains unidentified.

**Conclusions/Significance:**

These studies indicate that general and organ-specific metabolism is transformed in response to mitochondrial dysfunction, including digestive and absorptive functions, and give important clues as to how novel therapeutic strategies for mitochondrial disorders might be developed.

## Introduction

Human mitochondrial diseases affecting the oxidative phosphorylation (OXPHOS) system can result from a large number of different mutations, both in the nuclear genome or in the maternally inherited mitochondrial DNA (mtDNA) [1], [2]. Environmental factors can also trigger or aggravate these diseases. The clinical phenotypes of mitochondrial diseases are highly variable [2]. Although tissues most obviously dependent on bioenergy are commonly affected, notably heart and skeletal muscle, the central nervous system and sensory epithelia, the specific phenotypes are not understood.

In general, genetic disorders of mitochondrial OXPHOS can be classified into those affecting a specific subunit of one of the four OXPHOS complexes to which mtDNA-encoded translation products contribute, (equivalent to *mit^−^* mutations in yeast) and those affecting the biosynthesis of many or all of the mtDNA-encoded polypeptides (equivalent to *syn^−^* mutants in yeast). The former class includes disorders caused by point mutations either in mtDNA-encoded polypeptides, such as the NARP syndrome [3], or nuclear coded OXPHOS subunits[4]. The *syn^−^* class includes disorders such as MELAS or MERRF, caused by mutations in mitochondrial tRNA genes [5], as well as a diverse set of nuclear gene disorders caused by mutations in genes for the apparatus of mtDNA maintenance and expression. Examples of the latter include DNA polymerase γ [6], [7], mitoribosomal proteins MRPS16 and MRPS22 [8], [9], and the SURF1 assembly factor for complex IV (cytochrome *c* oxidase) [10].

In both arthropods and vertebrates, mtDNA is a compactly organized circular molecule which encodes just 13 of the more than 75 polypeptides that comprise the five OXPHOS complexes of the inner mitochondrial membrane. In addition, it encodes the two rRNAs and 22 tRNAs necessary for their synthesis within mitochondria. Mitochondrial protein synthesis also requires approximately 100 or more nuclear-coded gene products that have to be transported into mitochondria. In addition, all of the proteins involved in the maintenance, replication and transcription of mtDNA, as well as the many chaperones involved in the assembly of the OXPHOS complexes and the proteins that influence the intracellular organization and distribution of mitochondria are encoded in the nucleus [11], [12]. These nuclear encoded gene products include all of the protein components of the mitoribosome, which comprise an entirely different set than those present in cytosolic ribosomes [11]. Some of them have no counterparts in cytosolic or bacterial ribosomes, whereas others are phylogenetically conserved components of an ancient machinery of protein synthesis. One of these, the homologue of bacterial ribosomal protein S12, is a major component of the ribosomal decoding centre, and is of critical importance for translational accuracy. Mitoribosomal protein S12 (mRpS12) has been characterized in diverse taxa, including mammals [13], [14] and also *Drosophila* [13], [15], where it is encoded by the gene *technical knockout* (*tko*). It is well conserved in bacteria, as well as in the chloroplasts of higher plants and algae such as *Euglena*.

The gene name in *Drosophila* reflects the so-called bang-sensitive phenotype of the canonical allele, *tko^25t^*, which suffers paralytic seizures induced by mechanical stress, This phenotype is shared with other mutants affecting mitochondrial bioenergy supply, e.g. in genes such as *sesB*, the adenine nucleotide translocase [16], or *knockdown*, citrate synthase [17]. Null alleles of *tko* are larval-lethal, but the *tko^25t^* phenotype is relatively mild, and thus constitutes an animal model for mitochondrial disorders. In addition to seizure sensitivity, *tko^25t^* exhibits delayed larval development, antibiotic sensitivity, hearing impairment, locomotor hyporeactivity, and defective courtship [18]. It carries a point mutation, L85H, at a conserved amino acid of mRpS12, which leads to the destabilization or defective assembly of the small mitoribosomal subunit [14], [18]. The resulting insufficiency of mitochondrial translational capacity entrains a substantially reduced activity of the major OXPHOS complexes to which the mtDNA-encoded polypeptides contribute, both in larvae and in adults, which is believed to underlie the developmental and behavioural phenotype [18], [19]. All aspects of the mutant phenotype are restored to wild-type by expression of a transgenic copy of the wild-type *tko* gene under the control of its natural promoter [18]. The severity of the *tko^25t^* phenotype varies according to nuclear background [18] and gene dosage [20], indicating that compensatory mechanisms can partially alleviate the effects of this stress.

In order to gain insight into these compensatory mechanisms, and thus enhance our understanding of the global physiology of human mitochondrial disorders for which *tko^25t^* serves as a model [12], we carried out a transcriptome-wide analysis, using the Affymetrix platform. We identified a number of genes for components of metabolic pathways systematically up- (or down-) regulated at the RNA level, induction of some specific stress-response genes, alterations in the expression of certain genes involved in development and reproduction which mirror the organism-level phenotype, and increased expression of a number of genes putatively involved in intra- and intercellular signalling which suggest pathways by which these changes might be effected. Based on our findings, and extrapolating from *Drosophila* to humans, we suggest that nutritional supplementation might be considered an appropriate strategy in the management of some types of mitochondrial OXPHOS disease.

## Results and Discussion

### Identification of *tko^25t^*-Regulated Genes

**Figure 1:**
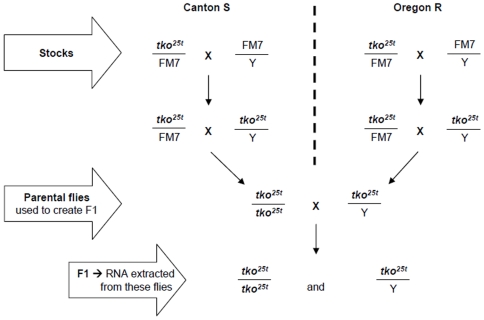
Crossing scheme to generate maximally outbred *tko^25t^* mutant flies for analysis. Balanced stocks were used first to create homozygous females and hemizygous males of the two parental backgrounds, in order to include in the analysis any maternal effects of the mutation. Note that *tko* is an X-chromosomal gene. The initial outbreeding to create the balanced stocks restores a wild-type genetic background, but does not completely eliminate any potentially compensatory recessive alleles already in the wild-type backgrounds. To minimize the effects of any such alleles, the crossing scheme illustrated is both maximally wild-type and heterozygous, under which conditions we saw the most substantial accentuation of the mutant phenotype, compared with inbred *tko^25t^* lines [115].

Inbreeding under stressful conditions inevitably results in the selection of compensatory alleles of many genes. Previous analyses of *tko^25t^* indicated that inbred lines were, indeed, subject to partial suppression of the mutant phenotype [18]. In order to avoid such issues, and thus determine the global effects on gene expression of the *tko^25t^* mutation in a truly unselected, ‘wild-type’ background, we outbred *tko^25t^* over more than 10 generations by back-crossing to each of two commonly used wild-type strains, Canton S and Oregon R. Subsequent to this backcrossing, *tko^25t^* was maintained in each background using a balancer chromosome. These stocks were then used to generate a *tko^25t^* mutant F1 generation, by crossing virgin Canton S *tko^25t^* homozygous mutant females with Oregon R *tko^25t^* males, as illustrated in Figure 1. For comparison, we generated otherwise isogenic wild-type F1 progeny by crossing virgin Canton S wild-type females with Oregon R wild-type males.

Flies of both sexes were collected from three independent such crosses, their RNA extracted and used to synthesize cRNA probes for hybridization to separate oligonucleotide arrays as described in Experimental Procedures. Data analysis for each gene in the array compared the signal of each *tko^25t^* mutant mRNA with the signal of each wild-type mRNA from flies of the given sex, over which the statistical analysis was performed. First of all, the data were prefiltered according to their detection p-value, selecting those probe sets with a significant p-value (<0.05) in their detection signal; this preliminary list comprised approximately 50% of the probe sets present in the array (Table 1). Afterwards, MAS5 and RMA algorithms were performed using SAM software in order to select those probe sets with significant differences in gene expression. Approximately 7% and 3% of the probe sets were picked with a fold change higher that 1.5, in males and females respectively (Table 1), although the false discovery rate (FDR) was very high (58% and 34% respectively). By increasing the stringency of the statistical analysis via extension of the cut off threshold (Δ-value) we reduced the FDR to less than 2.5%. After such filtering, 751 probe sets were identified as showing meaningful differences in expression between *tko^25t^* mutant and wild-type males, and 353 probe sets in females (respectively approximately 4% and 2% of the array).

**Table 1:** Number of selected probes during filtering and statistical analysis.

	number of probe sets		% of total a	
	male	female	male	female
Pre-filtering				
detection p-value <0.05	10110	9778	53%	52%
Filtering (SAM analysis)				
Fold change (R)>1.5	1248^b^	662^c^	7%	3%
both R>1.5 and FDR<5%	947	413	5%	2%
** both: R>1.5 and FDR<2.5%**	**751**	**353**	**4%**	**2%**

a % of probe sets in the array, to nearest whole number. GeneChip® Drosophila Genome 2.0 Array contains 18952 probe sets.

b FDR = 58%.

c FDR = 34%.

To gain an overview of the biological meaning of the differences in gene expression, probe sets corresponding to known genes were classified into different functional categories and pathways according to their gene ontology (molecular function, biological process and cellular component) as listed in Table S1. We then compared the output gene lists separately for male and female flies, which identified the categories systematically up- or downregulated in a sex-dependent and sex-independent manner (Table 2 and Figure S1). In mutant males, approximately the same number of genes was upregulated as downregulated. However, in mutant females upregulation was 3-times more prevalent than downregulation. About 25% of the genes upregulated in males were also upregulated in females, which corresponded to 38% of those upregulated in females. Nevertheless, most upregulated genes in the two sexes fell into the same functional categories or pathways (Table S2).

**Table 2:** Coherence of changes in gene expression by sex.

	Regulated genes^a^	% of regulated genes^b^	% of total genes^c^
male up (total)	404	54%	2.1%
male down (total)	347	46%	1.8%
female up (total)	268	76%	1.4%
female down (total)	85	24%	0.4%
both sexes up	102	14% (m), 29% (f)	0.54%
both sexes down	32	4% (m), 9% (f)	0.17%
male up female down	3	4‰ (m), 8‰ (f)	0.02%
male down female up	1	1‰ (m), 3‰ (f)	0.01%

a Number of genes regulated in the directional manner shown. For a graphical illustration see .

b % of the genes regulated in that sex.

c % of probe sets in the array. GeneChip® Drosophila Genome 2.0 Array contains 18952 probe sets.

Only about 9% of genes downregulated in males were also downregulated in mutant females, which nevertheless represented some 37% of those downregulated in females Table 2, Figure S1). However, many of the downregulated genes were already expressed in a sex-specific manner, and linked to reproduction. Setting these aside, the alterations to gene expression in *tko^25t^* mutant flies were qualitatively similar in the two sexes. Very few genes were oppositely regulated in the two sexes (less than 1%).

In the following sections we discuss the changes, classified according to biological process. The most highly up- or downregulated genes are listed separately in Table 3, with full details by functional category in Table S3. In a few indicative cases we validated the changes in expression using quantitative RT-PCR. In the following sections, the tissue-specificity of expression is based on www.flyatlas.org, plus other data cited in Flybase.

**Table 3:** Genes showing largest alterations^a^ in expression in *tko^25t^*.

Gene^b^	Function	FC (male)^c^		FC (female)^c^		Chromosomal localization
		Array	Q-PCR	Array	Q-PCR	
Bisexually upregulated genes						
*l(2)03659*	Mdr-related ABC transporter, xenobiotic clearance	43.0		11.7		45D1
*Fbp1*	Lsp receptor, aminoacid/nutrient transport	17.6	81	(33.7)	159	70D2
*Fbp2*	Lsp receptor, aminoacid/nutrient transport	16.5		(17.9)		30B3
*CG31775*	unknown function	12.2		(19.2)		35B5
*Obp99b*	odorant-binding lipohilic protein	13.6	2	(17.0)	17	99B8
*CG2650*	lipohilic hormone-binding protein	12.5		(18.0)		3B2
*CG17192*	gut-specific triacylglycerol lipase	9.6		17.9		97D14
*Cyp6a23*	cytochrome P450, xenobiotic metabolism	10.9		9.2		51D1
*Tequila*	serine protease	12.2		5.8		66F4
*CG11659*	long-chain fatty acyl-CoA synthetase	6.7	5	9.4	36	92B2
*Hsp22*	heat-shock protein	11.9	25	4.2	6	67B2
*CG3819*	endonuclease	5.7		10.4		75E6
*vav*	actin filament organization	14.1		(1.8)		18B6
*CG5999*	glucuronosyltransferase, xenobiotic metabolism	10.9		(3.9)		87C8
*Lsp1α*	aminoacid/other nutrient transport	7.2		(6.9)		11A12
*Cyp4e3*	cytochrome P450, xenobiotic metabolism	9.2		(4.8)		30C7
*nimC2*	unknown function	6.6		(7.2)		34E5
*Lsp1β*	aminoacid/other nutrient transport	6.3		(6.6)		21E2
*CG33346*	endonuclease	5.4		7.2		98E1
*CG12057*	unknown function	6.8		5.2		8C17
*CG15088*	sodium-dependent aminoacid transporter	4.6		7.3		55E10
*Lsp1γ*	aminoacid/other nutrient transport	6.0		(5.6)		61A6
*Peritrophin-15b*	gut-specific, chitin metabolism	1.9		8.4		29C1
*CG11893*	unknown function, protein-binding properties	6.1		(4.2)		96C9
*p24-2*	intracellular protein transport	3.7		6.4		85E4
*Ugt86Dd*	glucuronosyltransferase, xenobiotic metabolism	5.4		3.7		86D4
*CG5966*	triacylglycerol lipase	5.5		2.5		5D1
*Uro*	urate oxidase	(1.9)		5.7		28C3
*Jon25Bi*	gut-specific serine protease	(1.9)		5.7		25B4
*Ser6*	serine protease	5.8		1.7		19E5
*CG13947*	unknown function	3.4		3.4		21E2
*GstE1*	glutathione-S-transferase, xenobiotic metabolism/clearance	4.7		2.0		55C6
*Lsp2*	aminoacid/other nutrient transport	3.8		(2.7)		68F5
*unc-115*	actin-binding protein	3.4		3.1		85E4
*lectin-28C*	galactose-binding lectin	4.0		2.5		28D2
*CG13905*	unknown function	3.7		2.8		61D4
*Cyp6a8*	cytochrome P450, xenobiotic metabolism	3.3		3.1		51D1
*Jon25Bii*	gut-specific serine protease	(1.8)		4.5		25B4
*CG12780*	gram-negative bacterial binding	3.1		3.0		44D2
*CG11796*	4-hydroxyphenylpyruvate dioxygenase (aminoacid catabolism)	2.3		3.8		77C3
*CG1062*1	selenocysteine methyltransferase (aminoacid catabolism)	2.7		3.1		37B7
*CG31809*	steroid dehydrogenase	4.1		1.7		36B2
*Pepck*	PEP carboxykinase (GTP) 2	1.8	nt	4.0	2,5	55D3
*Fst*	positive regulator of fatty acid β-oxidation in response to cold	2.0		3.8		85E2
*ImpL3*	lactate dehydrogenase	2.6	1.5	3.1	4	65A11
*Hsp23*	heat-shock protein	3.4		2.1		67B3
*CG30016*	Malpighian tubule-specific steroid carrier	2.6		2.8		47C5
*Cpn*	calciphotin, calcium-binding, involved in eye development	2.3		3.1		87B1
*CG10592*	alkaline phosphatase, skeletal development	1.8		3.6		64D5
*εTry*	gut-specific serine protease	2.7		2.6		47F4
*CG30104*	nucleotide phosphatase	2.6		2.7		54B17
*Peritrophin-15a*	gut-specific, chitin metabolism	2.2		3.0		29C1
*CG5767*	unknown function	1.8		3.3		55B1–55B2
*CG3285*	sugar transport	2.8		2.2		23E4
*CG8942*	tenascin, Wnt-signalling receptor-related	2.1		2.9		34E5
*Cyp28a5*	cytochrome P450, xenobiotic metabolism	2.6		2.4		34E5
*Arc1*	cofactor for tRNA synthetase	2.5		2.5		50F6
Bisexually downregulated genes						
*RFeSP*	Rieske iron-sulfur protein, OXPHOS complex III (isoform A)	−27.3		−33.1		22A3
*gkt*	tyrosyl-DNA phosophodiesterase (DNA repair)	−5.4		−5.2		23D3
*HDC20470*	intergenic region	−6.1		−4.5		82A4
*dro4*	ion channel inhibitor with direct antimicrobial effect	−4.3		−4.3		63D1
*CG17478* d	ovary-specific unknown protein-binding protein (unlocalized gene)	−1.7		−6.8		41C1–41C6
*CG10924*	PEP carboxykinase (GTP) 1	−3.7		−(4.5)		55D1
*TotX*	humoral stress response protein	−5.0		−(2.0)		93A3
*phr*	deoxyribodipyrimidine photo-lyase (DNA repair)	−3.4		−2.8		43E18
*path*	aminoacids transporter	−2.1		−4.1		67B10
*CG11314*	mesoderm development	−3.2		−2.7		100A3
*CG10659*	unknown function	−2.9		−2.7		38B1
*Cyp6t1*	cytochrome P450, xenobiotic metabolism	−3.4		−2.0		20A1
Sex-specifically regulated genes						
*Sdic*	sperm-specific dynein intermediate chain	8.0		n.c.		19C1
*takeout*	lipohilic hormone-binding protein, behavioural regulator	−2.1	−5	n.c.	−2	96C7
*Gld*	glucose oxidase/dehydrogenase	−3.1		n.c.		84D3
*osk*	pole cell development	−8.8		n.c.		85B7
*CG12200*	unknown function, protein-binding properties	n.c.		15.7		18C7
*LysX*	gut-specific lysozyme	n.c.		7.0		61F3
*CG15533*	sphingomyelin phosphodiesterase	n.c.		6.4		99F4
*PGRP-SC1b*	defense against Gram-positive bacteria	n.c.		4.6		44E2
α*-Est10*	carboxyesterase	n.c.		−3.3		84D8–84D9
*bcn92*	mitochondrial-targeted, unknown function	2.2		−1.7		2D4
*Rala*	small GTPase	1.6		−1.9		3E5–3E6
*fit*	female-specific of *tra*	−3.0		1.9		93F14
Regulated transposable elements						
*Transposon.82*	transposon	22.3		10.2		---
*Transposon.11*	transposon	13.3		13.2		---
*Transposon.27*	transposon	8.9		7.4		---
*Transposon.17*	transposon	6.6		(2.5)		---
*Transposon.42*	transposon	2.7		2.4		---
*Transposon.30*	transposon	−2.3		−5.9		---
*Transposon.2*	transposon	−2.5		−3.2		---
*Transposon.22*	transposon	−5.6		−4.2		---
*Transposon.3*	transposon	1.7		−2.0		---

a Average of >2-fold change, both sexes considered, except in regard to genes with proven or probable sex-specific functions, where >2-fold change in only one sex was sufficient for inclusion in this list. For full list of alterations in gene expression, including details of relevant Affymetrix probe sets, see Table S3.

b Excluding genes normally expressed only in the opposite sex from that in which regulation was observed, or genes tightly inducible by a defined stress, e.g. bacterial infection, and which are normally expressed at a very low level in both sexes.

c Fold change, i.e. proportionate increase from wild-type (positive numbers) or to wild-type (negative numbers), in each sex. In parenthesis, those regulated genes that were unselected by the statistical analysis with the threshold that we used. Unaffected genes are denoted as no change (n.c.). Data shown alongside from the Affymetrix array experiment correspond to Q-RT-PCR analyses, where performed nt–not tested.

d Gene model currently withdrawn, probe set detects an ovary-specific transcript (flyatlas.org) at 41C1–41C6, but full genomic sequence not identified.

### Changes in Gene Expression Related to Metabolism

We observed systematically altered expression of genes concerned with energy metabolism, indicating a remodeling of metabolic pathways in response to the stress of mitochondrial OXPHOS insufficiency in *tko^25t^* mutant flies. Specifically, genes involved in the cytosolic reoxidation of NADH and in anaplerotic reactions feeding the TCA cycle, including amino acid and fatty acid catabolism, were upregulated, whereas those involved in conflicting pathways, notably fatty acid biosynthesis and the first steps of gluconeogenesis, were downregulated (Table S3-a). Although most of the changes in gene expression were quantitatively modest (typically 2-fold) the inferred pattern of global effects on metabolism is similar to that seen in yeast mutants with OXPHOS defects, via the so-called retrograde signalling pathway [21], [22]. We now consider in turn each of these inferred metabolic shifts, and the specific genes involved.

### Metabolic Shunts and Anaplerotic Pathways

At least two upregulated genes provide metabolic shunts for the regeneration of NAD^+^ from NADH, namely *ImpL3* (lactate dehydrogenase, which converts pyruvate to lactate), and *CG31674* (Table S3-a), the *Drosophila* orthologue of human glyoxylate reductase, which yields glycolate from glyoxylate. Pyruvate and glyoxylate may be considered major intermediate products of carbohydrate and amino-acid catabolism, respectively. Their diversion to essentially useless waste products (lactate and glycolate), which brings about the regeneration of NAD^+^, implies that carbon skeletons for biosynthesis normally derived from pyruvate or glyoxylate must be provided from other sources. This suggests a rationale for the upregulation of anaplerotic pathways and lipid/fatty acid catabolism. Another may be that, complex I being the component of the electron-transfer chain (ETC) quantitatively most affected in *tko^25t^* [18], breakdown of fatty acids by beta-oxidation partially circumvents the problem by feeding proportionately more electrons than pyruvate to the ETC at the level of complex III than complex I.

Unexpectedly, three enzymes of glycolysis, along with some other enzymes of glucose catabolism and transport, were down-regulated, but only in males (Table S3-a). However, in every case the down-regulated gene is the testis-specific isoform, with at least one other, much more highly expressed ‘ubiquitous’ isoform unchanged. These changes in gene expression most likely represent downregulation of the testis, and of reproductive functions in general, rather than being connected with any general transformation of metabolism.

The two isoforms of PEP carboxykinase (GTP) were reciprocally regulated. PEP carboxykinase (GTP) 1 (CG10924, downregulated in males along with pyruvate carboxylase) is the *Drosophila* orthologue of human PCK2, predicted to be mitochondrial and predominantly expressed in the larval fat body. PEP carboxykinase (GTP) 2 (Pepck, upregulated and widely expressed, closest homologue of human cytosolic PCK1 and also probably cytosolic despite its annotation [23]) is presumed to be the major anaplerotic source of oxaloacetate. The net effect is thus to activate a metabolic switch to spare the TCA cycle under conditions where pyruvate is mainly diverted to lactate.

### Lipid Metabolism

The two mRNAs for metabolic enzymes most dramatically upregulated in *tko^25t^* (CG17192, midgut-specific triacylglycerol lipase, Table S3-e, and CG11659, long-chain fatty acyl-CoA synthetase, most highly expressed in the Malpighian tubule, Table S3-a) both participate in the primary mobilization of dietary lipids. Another upregulated triacylglycerol lipase, CG5966 (Table S3-e), is widely expressed.

Some components of fatty acid beta-oxidation were upregulated (Tables S3-a, S4-e), such as beta-ketothiolase (*yip2*) and the ETF-ubiquinone oxidoreductase (*CG12140*), whereas enzymes of fatty acid biosynthesis were generally downregulated (see Tables S3-a and S3-e). However, many of these changes were only scored as statistically significant in one sex, since they were generally close to the filtering threshold of 1.5 fold.

### TCA Cycle and OXPHOS

Genes for TCA cycle components were generally not scored as changed in expression after statistical filtering, However, when we looked at them in the unselected data, (Table S4-a) there was a discernable pattern. Testis-specific isoforms were downregulated (in males), whereas many ubiquitously expressed isoforms were slightly upregulated (although this was usually below the filtering threshold and/or only in one sex). Perhaps surprisingly, only three (out of >50) nuclear-coded OXPHOS genes showed altered expression (Table S3-a). *CG10320 and CG33493*, encoding two subunits of complex I, were modestly regulated, but oppositely, and this was significant only in males. However, one of two mRNAs for *RFeSP*, encoding the Rieske iron-sulfur protein subunit of complex III, was downregulated more than 20-fold in both sexes. *RFeSP* is an essential gene [24], which generates two variant polypeptides by alternative splicing. The RFeSP-PB variant is more extensively homologous with the yeast orthologue Rip1p (Figure S2), whereas RFeSP-PA carries an unrelated C-terminus lacking some of the highly conserved Rieske domain. The *tko^25t^*-downregulated RFeSP-PA mRNA is normally expressed at approximately 20% of the level of RFeSP-PB mRNA, but in a similar tissue pattern. One possibility is that the former serves a regulatory role, e.g. in complex III assembly, although the exact reason for it being so strongly downregulated in *tko^25t^* is unclear.

### Protein and Amino Acid Metabolism

Many proteases and peptidases were induced in *tko^25t^*, some of them sex-specifically. Many are likely to be involved in the primary breakdown of dietary protein, since they are close homologues of gut-specific mammalian serine proteases such as trypsin and chymotrypsin [25] and are mainly expressed in the *Drosophila* gut, e.g. εTry [26], *Ser6* and at least ten members of the Jonah-family [27]. Upregulation of other proteases may serve a scavenging or recycling function, although that of Tequila (Table S3-n), *Drosophila* homologue of neurotrypsin [28], and elsewhere implicated in learning and memory [29], may be more connected with chitin metabolism. Released amino acids may provide an alternative source of carbon skeletons for biosynthesis, replacing pyruvate, less of which is entering the TCA cycle.

The sodium-dependent amino-acid transporter CG15088 (Table S3-f), as well as a number of enzymes of amino acid catabolism (Table S3-c), were upregulated in one or both sexes. Some enzymes annotated as being involved in amino acid biosynthesis were also upregulated, although their precise metabolic roles are unclear, as are those of many other enzymes, which may function in diverse pathways (Table S3-e). This complexity supports the idea that the mobilization of dietary protein mainly serves an anaplerotic rather than a purely catabolic role.

### Transport

The expression of genes for diverse transport functions was modified in *tko^25t^* flies (Tables S3-a and S3-f), most of which were increases. Focusing on the changes which were consistent between the sexes, and which were quantitatively the most dramatic, the major changes affect members of three families of sugar transporters, each expressed mainly in the Malpighian tubule and gut, hence implicated in dietary absorption, resorption or excretion. *CG3285*, *CG15406*, *CG7882* and 5 other upregulated genes are related to the yeast hexose transporter family (e.g. Hxt13p, Hxt2p) and to similar transporters in other eukaryotes and bacteria. *CG4726*, *CG8791* and three other upregulated genes belong to a family of sugar-phosphate transporters related to human SLC17A5, implicated in sialic acid storage disease [30]. *CG2196* and *CG8957* (plus three genes upregulated sex-specifically) belong to a family of ion transporters most closely related to the human sodium/glucose cotransporter SLC5A12. Two testis-specific sugar transporters, *Glut3* and *CG17637*, were downregulated.

Many other *tko^25t^*-upregulated transporter genes are expressed mainly or exclusively in the Malpighian tubule. *CG16727*, *CG8654* and *CG17752* (plus several other upregulated genes) belong to a superfamily of organic cation transporters, mammalian members of which are involved in diverse functions, including carnitine uptake [31] and excretion of xenobiotics [32].

*CG8323* (Table S3-a) encodes a member of the mitochondrial inner membrane carrier family. Its yeast orthologue Oac1p transports oxaloacetate, sulfate and hyposulfite. Enhanced capacity for oxaloacetate transport into mitochondria is consistent with the inferred anaplerotic function of Pepck upregulation. *CG18327, CG5805* and *Bmcp* are closely related members of the mitochondrial carrier super-family, most likely with overlapping substrate specificities and similar functions.

The changes in expression of genes connected with metabolism and transport in *tko^25t^* have features in common with those associated with other stress conditions, notably nutritional restriction [33] or starvation [34], as well as normal aging [33]. Under dietary restriction, many transport processes and extracellular functions, as well as fat body-specific genes and peptidases are upregulated. Some of the specific changes are seen also in *tko^25t^* flies, and many similar pathways seem to be affected. Furthermore, like *tko^25t^*, starvation induces the expression of genes involved in fat breakdown, fatty acid activation and beta-oxidation, as well as *Pepck*. Conversely, growth on sugar-rich diet rich induces a reciprocal pattern of changes, with up-regulation of biosynthetic genes for sugar to fat conversion, increased fatty acid anabolism and lipid biosynthesis, and downregulation of lipases. These findings suggest a common pathway of nutritional stress, involving signalling via one or a few key metabolites, and affecting genes for metabolic functions via a global response mechanism.

### Responses to Mitochondrial Stress

Other changes in gene expression appear to be specific to *tko^25t^*, suggesting a more direct response to failing mitochondrial protein synthesis. As shown in Table S3-b, genes for 12 mitoribosomal proteins, as well as a number of proteins involved in the processing of the mitochondrial translation products, were upregulated in *tko^25t^*. These include the mitochondrial prohibitin 2, *l(2)03709*, the *Drosophila* orthologue of the Rca1p m-AAA metalloprotease subunit, the mitochondrial deformylase CG31373, as well as a number of genes involved in the synthesis, mitochondrial import and processing of cytosolically synthesized proteins, notably chaperones Hsp10 (CG11267) and Hsc70-5 (Table S3-h). Strikingly, most of these changes were seen only in males. One possible explanation might be that many of the genes for mitochondrial biosynthetic components are highly expressed in ovary, being important in oogenesis, so that increased expression in somatic cells due to mitochondrial stress is not detected in females using the thresholds we employed.

The gene for the heat-shock protein Hsp22 was strongly induced (4–10 fold) in *tko^25t^* (Table S3-h). The gene is also upregulated during aging [35] and by oxidative stress. Mutations affecting *Hsp22* expression impair locomotor activity and result in decreased lifespan [36], whereas over-expression promotes resistance to oxidative stress and increases lifespan [37]. Another heat-shock protein of the lens alpha crystallin-related superfamily, Hsp23, was more modestly upregulated. A second group of stress-response genes upregulated in *tko^25t^* encode glutathione-S-transferases. At least 38 such genes are found in the *Drosophila* genome, of which 6 were significantly upregulated in either or both sexes in *tko^25t^* flies, although some others of them were repressed. These enzymes are required for the processing of oxidative adducts, such as peroxidated lipids, and their induction could be considered a signature of oxidative stress Three of them were previously found to be upregulated also by oxidative stress and/or in aging [38]. Mostly the upregulated genes of this class are larval, tubule or gut specific, whereas the downregulated members are testis or head specific.

One mitochondrial enzyme involved in iron-sulfur cluster assembly, cysteine desulfhydrase (CG12264, the *Drosophila* orthologue of yeast Nfs1p) was also upregulated. However, none of 25 genes arbitrarily selected from the NCBI GEO database, showing at least twofold induction by paraquat in *Drosophila* heads, were found to be also upregulated in *tko^25t^*. This indicates clearly that the pattern of changes in gene expression induced by severe oxidative stress is quite different from that seen in *tko^25t^*. It offers no support to the suggestion, based on other studies, that OXPHOS deficiency results systematically in ROS overproduction, and that the ensuing oxidative stress could constitute a common pathway of pathogenesis of mitochondrial dysfunction.

Glutathione-S-transferases are also considered to be physiologically important for the processing of xenobiotics for detoxification and excretion. Many other differences in gene expression in *tko^25t^*, whether scored as stress-related responses (Table S3-h), transport functions (Table S3-f) or endosomal-related (Table S3-i), could serve this same purpose. Glucuronosyltransferases such as UGt86Dd and CG5999, P450 cytochromes such as Cyp6a8, Cyp4e3 and Cyp6a23, and transporters of xenobiotics related to the mammalian multidrug resistance (Mdr) family, such as l(2)03659, are amongst the most highly induced genes. A set of induced lysosomal class II (degradative) alpha-mannosidases (CG9466, CG9463 and CG9468, Table S3-i) is likely also be involved in xenobiotic clearance. Increased mobilization of potentially harmful or non-metabolizable compounds may be a secondary effect of increased primary mobilization and absorption of dietary components. The induction of the above proteins would constitute a line of defense against such compounds, leading to their exclusion, activation, conjugation, and eventual excretion.

Another possible detoxification enzyme induced in *tko^25t^*, CG30022 (Table S3-h), is a mitochondrial member of the glyoxylase II superfamily and the *Drosophila* orthologue of *ETHE1*, the ethylmalonic encephalopathy disease-gene [39], whose manifestations include cytochrome *c* oxidase deficiency in muscle. The physiological role of ETHE1 is in mitochondrial sulfide detoxification [40]. Other members of the superfamily are implicated in clearance of metabolic by-products in cells with high glycolytic rates [41].

**Figure 2:**
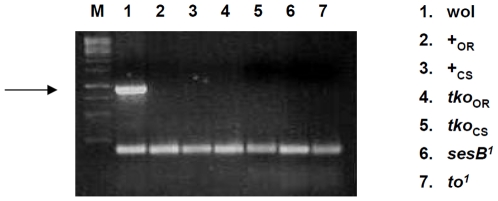
*Wolbachia* infection does not explain the abnormal metabolism or courtship behaviour of *tko^25t^* flies. PCR reactions analysed on agarose gels, using *Wolbachia*-specific 16S rDNA and *Drosophila* mitochondrial 12S rDNA primers. The 897 bp *Wolbachia*-specific product (arrowed) is detected only in the *Wolbachia*-infected strain obtained from the Bloomington Stock Center (wol), and not in wild-type (+) or *tko^25t^* flies in either the Oregon R (OR) or Canton S (CS) backgrounds, nor in inbred laboratory stocks of the *sesB^1^* or *to^1^* mutants. The 180 bp mitochondrial DNA product is evident in all strains tested. M, 1 kb marker ladder.

Several genes related to the innate immune response were upregulated. To test whether this reflects a response to possible chronic infection of the *tko^25t^* stock with *Wolbachia*, which might also contribute to the abnormal reproductive behaviour of *tko^25t^* males [42] we performed PCR using *Wolbachia*-specific 16S rDNA primer pairs. This failed to detect any evidence of infection (Figure 2). A second possibility is that the induction of antimicrobial defense genes is due to activation of a common signalling pathway involved in stress responses. A third possibility is that metabolic stress may render the organism more susceptible to infection, and priming of key defense mechanisms may be advantageous to survival under such conditions (or could be a response to the actual proliferation of commensal bacteria). Most antimicrobial peptides are tightly inducible by pathogen challenge [43]–[45]. Their low level of expression in wild-type flies means that many of the largest changes (such as 8-fold upregulation of *Defensin* in males, 2-fold in females) did not pass the statistical thresholds. Some (e.g. dro4) were also down-regulated.

Two *tko^25t^*-upregulated stress-related genes, *adipose* and *Frost* (Table S3-h), are involved in metabolic responses to other environmental stresses [46], [47]. The action of the adipose protein appears to be in the storage, transport and metabolization of triacylglycerol, [48] which fits the general pattern of induction of genes involved in the dietary mobilization of fats. Mutants suffer hypertrophy of the fat body, due to the accumulation of fat droplets [49], although the protein is widely expressed. Fst is expressed mainly in gut and tubule and its metabolic functions are not clear. It is also modulated by bacterial infection of the gut [50]. CG17734, upregulated in males (also in females by 40%, hence missed in filtering) is a homologue of the mammalian, mitochondrially localized HIG1 (hypoxia-inducible gene) family, which protects cells from apoptosis under conditions of hypoxia or glucose deprivation [51].

### Effects on DNA and RNA Metabolism

Systematic effects of *tko^25t^* on nucleic acid metabolism were relatively few (Table S3-d). There was a pronounced upregulation of gut-specific DNA endonucleases CG3819, CG33346 and CG6839 (females only), possibly indicative of the mobilization of additional dietary components. Two (out of the many) genes connected with DNA repair were downregulated in both sexes. *gkt* (*glaikit*, *Tdp1*) is annotated as tyrosyl-DNA phosphodiesterase, an enzyme involved in the resolution of ‘dead-end’ complexes' between DNA and topoisomerase I [52], as well as in other DNA repair pathways [53]. However, *gkt* mutants also show neural phenotypes associated with deranged epithelial cell polarity [54], proposed to be due to the lack of phospholipase activity of the gene product. *phr* (*photorepair*) is responsible for the repair of UV light-induced cyclobutane-type pyrimidine dimers [55]. The significance of these changes is unclear.

### Cell Cycle Regulation, Development, and Cell-Death

Logically the developmental delay and reproductive phenotypes of *tko^25t^* should be reflected in subtle alterations in the expression of developmentally regulated genes, in particular those connected with metamorphosis, organogenesis, and reproduction. Many changes in gene expression indeed fell under these headings (Tables S3-g, S3-j, S3-k and S4-b). However, they are hard to interpret unambiguously, since the vast majority affected only one sex, and only in rather few cases were multiple genes contributing to a single organ, differentiation programme or physiological process clearly coregulated.

There was substantial upregulation in *tko^25t^* of a set of genes expressed in the larval fat body, equivalent to the mammalian liver, and encoding the major larval serum proteins Lsp2 (3–4 fold) and all three subunits of Lsp1 (6–7 fold, Tables S3-g, S4-b). Upregulation was similar in males and females, but seems not to have passed statistical filtering in females, due to the very low expression in wild-type adults [56]. The upregulation of *Fbp1* and *Fbp2*, considered as receptors for the larval serum proteins, was even more substantial (up to 2 orders of magnitude), and that of *Fbp1* was verified in both sexes by Q-RT-PCR. The upregulation of these genes may represent the persistence of larval gene expression connected with developmental delay. Their expression in *tko^25t^* adults is at least an order of magnitude less than in wild-type L3 larvae, when the genes are most highly expressed.

These various proteins have been proposed to play roles in wound healing, nutrient transport, oxygen diffusion and immunity. Their stage-specific regulation [57], [58] suggests that they provide a nutrient storage system during metamorphosis, involving resorption of the serum proteins into the fat body at L3 stage [58]. The circulating serum proteins may also serve a more general nutrient transport function in larvae and adults. Their upregulation in *tko^25t^*, as under dietary restriction [33], might contribute to more efficient absorption of dietary components or clearance of xenobiotics, involving their transport to the fat body for detoxification and eventual excretion. Some unrelated serum protein genes were also upregulated, including *fat-spondin, Idgf5*, two monooxigenases and one endopeptidase (*CG3505*), implicated in clotting [59] and cuticle formation (Table S3-k).

Upregulation of genes connected with skeletogenesis [60], [61] (Table S3-k), including constituents of the cuticle, proteins involved in chitin metabolism, and several alkaline phosphatases, some of them gut-specific, may again be a signature of delayed development. Changes in the expression of some genes normally expressed only at very early developmental stages can probably be disregarded as quantitatively trivial (Table S3-k), although the repression of genes involved in sense organ development such as *Optix, mirr, phl, sdk, Magi, ana, Oseg1,Tig* and *nompB* (generally significant only in males), could be related to the sensory deficit seen in *tko^25t^* flies.

The apoptosis and autophagy pathways were generally unaffected, perhaps surprising given the fact that nutrient deprivation induces autophagosome upregulation in many organisms [62], [63]. One obvious explanation would be that increased mobilization of dietary resources is sufficient to overcome the metabolic consequences of the mutation.

**Figure 3:**
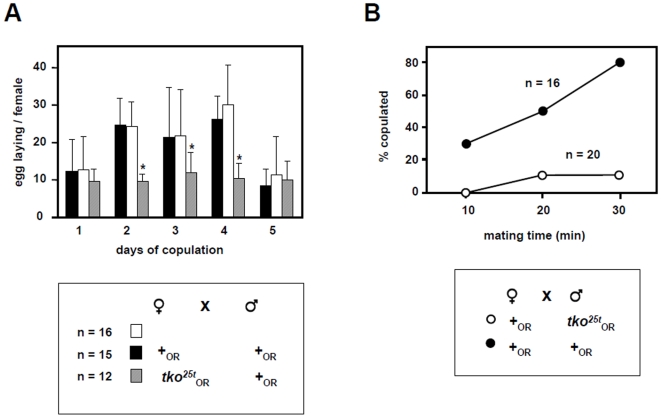
Reproductive defects of outbred *tko^25t^* females. (a) The number of eggs laid per mated female was counted daily for individual mated females from the crosses indicated. Asterisks indicate significant differences (*p*<0.01, *t*-test). (b) Single mating pairs of the genotypes shown were observed for time to copulation.

Changes affecting cell division and cytoskeletal functions (e.g. *unc-115, vav*, Table S3-j) are hard to interpret. The impacted pathways are again similar to those induced by dietary restriction [33], although many of the affected genes are different. The downregulation of histones and of genes involved in the cell division apparatus may be an indicator of decreased cell division in the germline. For example, the downregulated gene *piwi* (Table S3-m) promotes cell proliferation and differentiation in the female germline [64]. The implied decrease in female gametogenesis was confirmed by measuring oviposition of *tko^25t^* females outbred into the Oregon R background, when mated to wild-type males. The total number of eggs laid by *tko^25t^* females over 5 days was approximately half the number laid by wild-type females (Figure 3a).

Strikingly, about two-thirds of the many genes of unknown function downregulated specifically in males (Table S3-o) are expressed specifically in testis. This fits with the idea that mitochondrial dysfunction, perceived as a nutritional limitation, provokes a general shift of resources away from reproduction towards maintenance. Curiously, however, the same does not appear to be true of females, where rather few downregulated genes are ovary-specific. Most of the functionally unidentified genes downregulated only in females (Table S3-o) show widespread expression, whereas unidentified genes upregulated only in females are mainly gut-specific.

### Expression of Other Sex-Specific Genes

Many of the genes regulated differently between the sexes in *tko^25t^* (Tables 3, S3-m, S4-c) are already expressed sex-specifically, being putatively involved in gametogenesis, sex determination or reproductive behaviour. However, some appeared to be downregulated in the sex where they are not usually expressed, which can be considered to have little or no physiological meaning.

In the sex determination pathway (Figure S3), both sexes showed evidence of feminization (Table S3-m): the female-specific *doublesex* transcript (dsx^F^) was upregulated in females, as was *fit* (*female-specific independent of transformer*), which may itself be regulated by the dsx^F^ product. Downregulation of *fit* in males is probably inconsequential, since it is normally expressed in males only at a low level. However, males also showed downregulation of several genes implicated in regulation of male-specific functions, notably *takeout* (*to*) and *sxe2* (*male sex-specific enzyme 2*).

The product of *takeout* belongs to an insect-specific family of lipohilic ligand-binding proteins, the best characterized member of which is the juvenile hormone binding protein of *Manduca sexta*. They appear to influence various behaviours and developmental events, and are expressed in response to diverse signals. *takeout* expression shows cycling under the control of circadian regulators and is upregulated by starvation, to which *to^1^* mutant flies are hypersensitive [65]. *takeout* mRNA is expressed in a highly localized manner in structures within the gut and the antennae [65], as well as male-specifically in the adult fat-body. The protein is widely distributed, though its presence in the hemolymph is male-specific [66]. It has been proposed to regulate both feeding and reproductive behaviour [67], and in males promotes (and is required for) courtship [66], [68]. Its basal expression level is influenced by *dsx* [68] and by *fruitless* (*fru*), and the *to^1^* mutation results in behavioural feminization either in an outbred genetic background or in *fru* heterozygotes [68].

**Figure 4:**
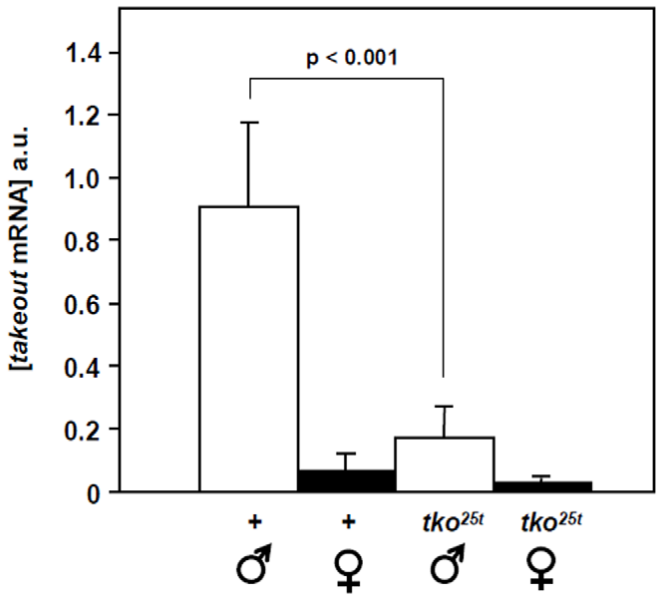
Quantitative RT-PCR verification of transcriptomic data on *takeout*. RNA measurements were made and normalized as described in Materials and Methods. Means±SD of three sample runs of each of three biological replicates are shown. Significance at the *p* value shown was computed using a *t*-test. See also Table 3.

*takeout* downregulation thus provides a plausible explanation for the male courtship defect of *tko^25t^* males, which we verified in flies outbred into the Oregon R background (Figure 3b). In wild-type flies the circadian cycling of *takeout* mRNA exhibits a peak-to-trough ratio of approximately 5, and the decrease in *takeout* mRNA levels seen in *tko^25t^* males (Figure 4), may simply reflect a loss of this cycling. Since starvation induces *takeout* expression in flies not synchronized in a light-dark cycle [65], a disturbance in circadian cycling of *takeout* might stimulate food-seeking behaviour whilst suppressing male courtship. This makes biological sense, delaying reproduction under conditions of limited food resources.

*CG2650*, upregulated 12-fold in males (Table S3-n), and also in females (though excluded by statistical filtering), encodes an RNA highly expressed in the last stages of pupation under circadian control, and localized to the cuticle of the newly eclosed adults [69]. Its level decays rapidly following eclosion, hence the upregulation in *tko^25t^* may be considered a further example of developmental delay. *CG2650* is a member of the same gene family as *takeout*. The 3′ untranslated portion of the *CG2650* mRNA overlaps that of the circadian regulator *per* by 60 nt, suggesting possible mutual regulation by RNA interference. One possibility is that *CG2650* upregulation disrupts expression of *per*, leading to the male-specific downregulation of *takeout*; another is that temporally altered expression of these putative hormone-binding proteins is induced by persistence of juvenile hormone.

The odorant-binding protein gene *Obp99b* (*tsx*), normally expressed more highly in males than in females under the regulation of *dsx* [70], was highly upregulated in both sexes: although excluded by statistical filtering in females, Q-RT-PCR showed a much larger elevation in females than in males. This may represent an additional mechanism to attenuate reproduction under unfavorable conditions, since ectopic *Obp99b* expression in females negatively regulates receptivity [70], [71].

### Signaling

**Figure 5:**
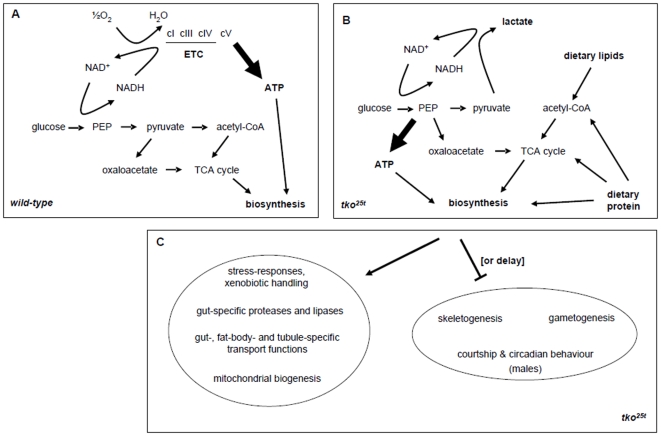
Summary of major alterations to gene expression and their proposed effects in *tko^25t^* flies. (a, b) Proposed metabolic effects, based on differences in gene expression affecting nutrition and metabolism between (a) wild-type and (b) *tko^25t^* flies. In wild-type flies glucose is metabolized via PEP to pyruvate, which is then fed to the TCA cycle mainly via the pyruvate dehydrogenase complex generating acetyl-CoA, with a small amount converted to oxaloacetate to replenish the TCA cycle intermediates as needed, maintaining a supply of carbon skeletons for biosynthesis. Surplus NADH is reoxidized via the ETC (complexes I, III and IV), generating potentially most of the cell's ATP needs at complex V. In *tko^25t^* flies, the maximal activity of the ETC complexes is only 10–20% that of wild-type flies [18]. For simplicity, its greatly decreased contribution to NADH oxidation and ATP generation is omitted altogether in panel (b). Instead, the bulk of ATP must be supplied by glycolysis, with NADH reoxidation dependent on lactate dehydrogenase and similar shunts. Because pyruvate is, under such conditions, mainly shunted to lactate, the TCA cycle must be supplied from other sources, via the mobilization of dietary lipids, generating acetyl-CoA, PEP carboxykinase (I) diverting a small amount of PEP to oxaloacetate, and the mobilization of dietary protein and amino acid catabolism supplying these and other TCA cycle intermediates, as well as biosynthetic reactions directly. The modifications to metabolism in *tko^25t^* flies are accompanied (c) by altered expression of genes connected with nutrient breakdown, absorption and transport, plus xenobiotic handling, affecting mainly the gut, Malpighian tubule and fat body. In addition, there is downregulation or delayed expression of genes connected with gametogenesis and skeletogenesis, and, notably in males, altered expression of genes controlling circadian and courtship behaviour, interpretable as a biological response to poor nutritional conditions.

In this study we found several types of genes to be regulated in a systematic way, indicative of a signalling pathway that senses mitochondrial stress and generates specific transcriptional readouts (summarized in Figure 5). Few known molecules involved in signalling were themselves transcriptionally responsive in *tko^25t^*. To widen the search for relevant signalling pathways we looked also at their reported interaction partners and those of other plausible candidates.

The serine-threonine protein kinase *Akt1*, known to exhibit a plethora of developmental and physiological signaling functions [72], [73], including responses to nutritional conditions [73], [74], was upregulated too modestly to pass statistical filtering. Of 161 gene entries for Akt1 in the DroID interactions database (www.droidb.org), only 9 were significantly *tko^25t^*-responsive, close to random expectation. Apart from the putative spliceosomal component CG13900, downregulated in females only, and nucleosome positioning protein Nlp, downregulated in both sexes, all were regulated in males only, but in either direction. Although CG13900 is also downregulated in larvae under starvation [34], the evidence that Akt1 is involved in signaling of mitochondrial stress and metabolic adaptation in *tko^25t^* is, at this point, purely speculative.

*shaggy* (*sgg*), upregulated in *tko^25t^* males, encodes a serine-threonine protein kinase with a widespread expression pattern and involvement in many metabolic, behavioural and developmental processes [75], some of them relevant to the *tko^25t^* phenotype, including sense organ specification [76] and circadian and courtship behaviour [77], [78], [79]. Its mammalian homologue, GSK3 [80] plays a key role in cellular signalling cascades, and interacts with PKB (homologue of *Akt1*) [81], [82]. In *Drosophila*, the phosphorylation of tim by sgg promotes its translocation to the nucleus, driving the circadian pacemaker [77], and suggesting a pathway for the regulation of *takeout*, which is a known target of *tim*. A further interactor of *tim*, the circadian regulatory transcription factor *Clk*, was 2-fold upregulated in *tko^25t^* females, although this failed statistical filtering (Table S4-d).

I-2 (Table S3-l), downregulated (but only significant in females), is a widely expressed protein phosphatase inhibitor. Inhibition of protein phosphorylation produces pleiotropic developmental phenotypes via the antagonization of proliferative signals [83], but there are no known links with nutritional or stress-response pathways. Two (of 14) reported interaction partners of I-2, Pp1-13C (Table S3-l and testis-specific) and sgg were regulated, but only in males, and in opposite directions.

*Tsp42Ed*, a gene encoding a protein of the tetraspanin family was found to be upregulated in *tko^25t^*, but this also was significant only in females Tetraspanins are expressed in distinct tissue and developmental patterns and are functionally diverse, having roles in cell migration, signaling, cell fusion and adhesion [84]. Tsp42Ed is highly expressed in tubule, gut and fat body, but its specific role in signaling, if any, is unknown.

In yeast, the retrograde pathway depends upon three gene products without convincing structural homologues in metazoans. Rtg3p and Rtg1p are zinc-finger transcription factors distantly related to Mitf and Usf, but having quite different functions. The sensor protein Rtg2p has homologues in fungi but not beyond, and its ligand remains unidentified. It is distantly related to bacterial polyphosphatases, which are stress markers, especially of amino acid starvation [85]. Responses to amino acid starvation in both yeast and higher eukaryote involve the Target Of Rapamycin (TOR) pathway [86], with Akt as a downstream target. In yeast, the RTG and TOR pathways interact. No components of the *Drosophila* TOR complexes (*Tor*, *raptor*, *rictor*, *Sin1*, *CG3004*/*Lst8*,) were *tko^25t^*-responsive, nor were any of their known or predicted targets (*S6K*, *Thor*). We looked also at their interaction partners, but here again only 12 new targets of *tko^25t^*-regulation were identified, out of >200 such proteins, and all such cases were predictions from yeast rather than genetically or biochemically proven interactions. Only one of them, CG10592, was significantly (up)regulated in both sexes. However, this and 5 others represent a coherent set of proteins involved in skeletogenesis.

Neither CG9809 (Spargel), the *Drosophila* orthologue of PGC1α, the proposed global regulator of mitochondrial biogenesis in mammals [87], nor its interaction partners CG15323 and CG7800, were *tko^25^* ^t^-responsive. However, 9 out of 142 predicted or known interaction partners of *SNF1A*, the *Drosophila* homologue of *AMPK*, implicated in the regulation of lipid metabolism in mammals, showed altered expression, and the overall readout of changes in fatty acid metabolism and Pepck (CG10924) [see 88] suggests its possible involvement. Four of the 9 regulated genes are members of the cytochrome P450 superfamily, some of which are known to be regulated via AMPK in mammals [89], [90]. SNF1A itself was unaffected. Another possible candidate for involvement in retrograde signalling in *tko^25^* ^t^ is CG17734, a homologue of HIG1 [91], which upregulates glycolysis and anaerobic metabolism in mammals, and which was induced almost 2-fold in males (Table S3-h).

To identify possible hormonal signals mediating downstream metabolic responses, we searched the gene list for putative peptide hormones, peptidases and enzymes which could be involved in ecdysteroid or sesquiterpenoid metabolism, including those without clearly established physiological roles (Table S5). We considered only those which responded in both sexes, and were expressed specifically in the larval fat body and/or adult head (where the fat body is located). Excluding metabolic enzymes with no known roles in steroid or sesquiterpenoid biosynthesis, we narrowed the gene list to three candidates, none of which is compelling.

Two of them encode proteins related to antimicrobial responses. *dro4* (drosomycin-4, 4-fold downregulated) is one of a cluster of seven putative antifungal peptide genes [92], [93], also expressed at a substantial level under standard conditions. PGRP-SD (2-fold upregulated in males is involved in the recognition of gram-positive bacteria [92]. One possibility is that it is targeted against bacteria which produce mitochondrial toxins, such as *Streptomyces antibioticus*, which secretes antimycin A. Upregulation of PGRP-SD may thus be directed against an unseen pathogen.

TotX is a member of the Turandot family of humoral peptides [94], induced by various stresses, and believed to mediate repair processes. It is strongly induced by bacterial infection or by paraquat [94]. However, TotX is downregulated in *tko^25t^*, and the change was not scored as significant in females.

In summary, despite some intriguing circumstantial evidence, we found no convincing data to support the involvement of any known intracellular or humoral pathway in mediating responses to mitochondrial stress in *tko^25t^*. Clearly a different experimental approach will be needed to reveal any such pathways and their components.

### Other Regulated Genes

Expression of a small number of genes implicated in behaviour, in addition to those directly involved in courtship, was found to be altered in *tko^25t^* (Tables S3-n and S4-d). These include *no extended memory* (*nemy*, mitochondrial glutaminase), whose upregulation might be part of the retrograde response to maintain glutamate levels anaplerotically, and *Rhythmically expressed gene 2* (*Reg-2*) a haloacid dehydrogenase, perhaps also with a metabolic function. Apart from *Obp99b*, the expression of several odorant-binding protein genes [95] was modified sex-specifically, although the genes remain functionally unclassified in regard to ligands or downstream effects. Some may play roles in pheromone perception and mating behaviour, since many of the same genes were differently expressed in flies selected for fast *versus* slow latency in mating [79]. Some other genes picked up in the latter screen were also affected by *tko^25t^*, including *sgg*, *Clk*, *tim*, *Dat*, *elav* and *Hsp27* [79].

One possible explanation for the regulation of genes without obvious biological sense is that such genes may be coexpressed with nearby genes whose regulation is of functional importance, with which they share *cis*-regulatory elements. To investigate whether the altered expression of any genes in *tko^25t^* might be attributable to such an effect, we compared the chromosomal locations of the genes listed in Table 3. Other than co-regulated, adjacent members of the same gene family (Hsp22/Hsp23, Jon25Bi/ii, Peritrophin-15a/b), plus *unc-115* and *p24-2*, which are adjacent at band 85E4 and possibly involved in the same cellular process, we saw no plausible examples of such opportunistic regulation.

The list of most highly regulated genes (Table 3) includes 11 genes with no annotated function. Of these, nimC2 (larval fat body-specific) is almost certainly involved in phagocytosis, like its homologues Eater, Draper and nimC1 [96]. The putative gene product of *CG31775*, also larval fat body-specific, is a bizarre short polypeptide with oligoglutamine, glycine and alanine repeats. These make it impossible to identify homologues by conventional search programmes and it is far from certain that it is ever translated. *CG13947* (salivary gland and crop-specific) also has an internally repetitive structure and is practically impossible to parse for similar reasons. *CG12057* and *CG5767* (upregulated) and *CG10659* (downregulated) are all midgut-specific. The first two have homologues only in the Drosophilids, and their function is completely unknown, but CG10569, with homologus in diverse taxa, has an acyl-CoA N-acyltransferase domain, although its substrate(s) are unidentified. CG13905 (tubule-specific) is found in other insects and is induced by other stresses, notably by pathogens. CG12200, CG17478 (both ovary specific) are annotated as having protein-binding properties. The apparent regulation of the latter in males can be discounted. CG12200 (strongly upregulated) is a RING finger protein with weak homologues in many taxa and may be assumed to be involved in protein metabolism. CG11893, another upregulated gene suggested to have protein-binding properties and possible protein kinase function, is very weakly expressed in many tissues and its function cannot be extrapolated. Lastly, *bcn92* (*CG3717*), annotated as mitochondrially targeted, and a rare example of a protein significantly regulated between the sexes in a reciprocal manner (up in males, down in females) is related to proteins involved in iron-sulfur biogenesis (Isd11p in yeast) and complex I subunit NDUA6, and is probably the orthologue of the former.

Finally, the *tko^25t^*-regulated gene list includes 12 transposable elements, 266 functionally unclassified genes (117 upregulated, 137 downregulated), as well as 44 Affymetrix probe-sets currently assigned to intergenic regions (Table S3-o). Only around 10% of them appeared to be similarly regulated in the two sexes.

### Congruence with Gene Expression in Other Models of Mitochondrial Disease

Mitochondrial diseases in humans are physiologically and biochemically diverse, and *tko^25t^* should be considered a strict model only for those cases where there is a generalized defect of mitochondrial protein synthesis. However, this includes the common disorders associated with mtDNA deletions or tRNA point mutations, as well as an increasing number of nuclear genome-determined disorders.

Studies of gene expression in *Drosophila* essentially track changes in the whole organism. This may bias the results in favour of genes expressed prominently in major organs, although regulation specific to the needs of a single tissue is less likely to be mistaken for a general response pathway. The latter is a particular problem when studying individual mammalian cell-lines, whose developmental origin or tumorigenic nature may bias the set of affected genes. For example, a recent analysis of gene expression changes associated with OXPHOS dysfunction in the 143B osteosarcoma cell background [97] identified a number of targets involved in invasiveness, notably upregulation of MMP1 and downregulation of its inhibitors. Although revealing an unexpected role for OXPHOS dysfunction in bone cancer, the wider relevance to OXPHOS dysfunction is questionable. Conversely, substantial changes in expression of tissue-specific genes in *tko^25t^*, e.g. those expressed in gut or Malpighian tubule, are almost certainly of global physiological significance, but would be invisible in cultured cell studies, unless a wide range of cell-types was studied.

In order to minimize such problems, Alemi *et al.* [98] studied a variety of different cell types bearing pathological mtDNA deletions and looked for coherent patterns of altered gene expression. Consistent changes occurring in at least 4 of the 7 cell-types studied were considered meaningful. However, since only one *in vivo* tissue was included, the results remain strongly biased in favour of changes seen in cultured (cancer) cells, which typically revert to a highly glycolytic metabolism, relying on OXPHOS mainly for specific biosynthetic pathways such as pyrimidine biosynthesis, where the ETC performs a redox role rather than acts a source of ATP. Despite this, the genes most highly responsive to mitochondrial dysfunction were different from those identified in the 143B cell study [97], apart from the upregulation of the key autophagy gene ATG12, syntaxin-16 and of the Hsp70 family member HSPA4.

In this light, it is not surprising that there is little congruence between our gene list and those of previous authors, or between those of previous authors when compared with each other. It must be kept in mind that the physiology of the whole organism is a co-operative enterprise between different organs with complementary functions, whose responses to mitochondrial dysfunction may also be reciprocal, generating a superficially contradictory picture when gene expression in the whole organism is tracked. We analysed the behaviour in the *tko^25t^* model (Table S6-a) of the closest *Drosophila* homologues of the 222 genes scored as showing consistently altered expression in 143B cells under conditions of OXPHOS deficiency [97]. Seven showed significantly altered expression in *tko^25t^*, close to random expectation, most of them only in one sex, or in the opposite direction to that seen in 143B cells. However, fewer than half of the total gene list has a convincing orthologue in *Drosophila*, which means that some of the congruent changes could be meaningful. Two of them showed indisputable orthology and similar regulation in all 143B cell models plus both sexes in *tko^25t^*: *CG3961* (*ACSL1* in human) and *unc-115* (*ABLIM1* in human), both of which were upregulated (although for *CG3961* this was significant only in males). *ACSL1* (*CG3961*, long-chain fatty acyl-CoA synthetase) is the key enzyme of fatty acid mobilization. Its upregulation in OXPHOS-deficient 143B cells may serve the same anaplerotic function we hypothesize in *tko^25t^*. *ABLIM1* (*unc-115*) encodes a cytoskeletal actin-binding protein [99]. Its exact physiological functions are unclear. Its homologue in *C. elegans* regulates the formation of lamellipodia and filopodia in axonal guidance, and one isoform performs a similar role in *Drosophila*. However, *unc-115* is widely expressed, and may play a more general role in downstream signalling from Rac. One of its (few) predicted interaction partners, *drk*, is a signal transduction molecule involved in development of the peripheral nervous system [100], which may be relevant to the sensory defect of *tko^25t^*.

Comparing gene expression alterations in *tko^25t^* with the study of mtDNA deletions in different cell types [98] we saw almost no overlap (Table S6-b). Many OXPHOS genes were downregulated in the deletion study, but few or none in *tko^25t^* or 143B cells. Unlike the deletion study, we saw no systematic downregulation of the ubiquitin/proteasome system, nor any general upregulation of vesicle transport, although we did see a strong upregulation of p24-2, involved in this process and expressed widely [101] but most prominently in the salivary gland. Apart from the modest downregulation of the NDUFB3 subunit of complex I in males (*CG10320*), and upregulation of a subunit of the vacuolar ATPase, the only congruent observation was upregulation of *GLS* (*nemy* in *Drosophila*), interpreted as part of a general enhancement in amino acid catabolism as in our own study. The involvement of glutaminase is intriguing, since glutamate has been postulated as a key intermediary metabolite sustained by the retrograde response in yeast [22]. In the three studies there are distinct alterations in protein metabolism: the induction of gut-specific proteases and enzymes of amino acid catabolism in *tko^25^*, proteasome downregulation in cells with mtDNA deletions, and the activation of autophagy in OXPHOS-deficient 143B cells. All three may constitute a response to mitochondrial dysfunction perceived as amino acid starvation, although their physiology differs.

Comparing our findings with the one substantial study of gene expression in an *in vivo* mammalian model of mitochondrial dysfunction [102] reveals both similarities and also some striking differences (Table S6-c). The latter study compared gene expression in hearts of mice in which a tissue-specific mtDNA depletion was induced by heart-specific knockout of the *Tfam* gene. In the intermediate and late stages of the resulting cardiac-disease there was a consistent shift in the pattern of expression of genes connected with metabolism. However, although many of the same genes and pathways were affected as in *tko^25t^* (TCA cycle and fatty acid catabolism), mostly they were oppositely regulated, and the change in expression in *tko^25t^* was so modest that it was rarely scored as significant in our filtering (Tables S4-a, S4-e). Some glycolytic enzymes were also upregulated in *Tfam*-knockout hearts, whereas we saw an increase only in LDH and other shunts for regeneration of NAD^+^.

Similar to our findings, no substantial or systematic differences were seen in the expression of nuclear genes for OXPHOS subunits, whereas some genes involved in mitochondrial biogenesis were upregulated, including the mitochondrial chaperones Hspa9 and Hsp1 (Hsc70-5, CG11267) and a set of mitochondrial ribosomal proteins partially overlapping with those picked up in our own study. The most prominently upregulated gene in the *Tfam*-knockout heart, the mitochondrial Lon protease Lonp1 (*CG8978* in *Drosophila*), was unaffected in *tko^25t^*. A more detailed comparison with the *Tfam* knockout study is not possible, since Hansson et al. [102] did not publish their data relating to other genes than those discussed here.

Signalling pathways implicated in the response to mitochondrial dysfunction appeared to differ in all four studies. The presence of mtDNA deletions induced up-regulation of the AMP-activated protein kinase (PRKAA) and its targets [97], [103], with cellular ATP depletion postulated as the primary signal. In 143B cells study about 20% of all genes affected were connected with signaling, although no single pathway was implicated. Many genes responding to *Tfam*-knockout are regulated by PPARα [102]. In *Drosophila*, we found only weak evidence implicating any signalling pathway, although Akt1/sgg and AMPK seem the best candidates.

The differences in the *Tfam* and *tko^25t^* models may reflect the specific physiology of the heart which, in *Drosophila*, as the dorsal vessel [104], [105], accounts for only a tiny fraction of the total tissue mass. Another difference is that *Tfam* knockout progressively abolished OXPHOS function whereas it is only downregulated by *tko^25t^* [18]. Finally, the changes in the *Tfam*-knockout heart might reflect tissue remodeling, rather than a shift in gene expression of a specific cell-type, as suggested by the gross anatomical changes seen in the failing heart: *tko^25t^* causes no major anatomical abnormalities.

### Implications Regarding Management of Mitochondrial Disease

The primary response to mitochondrial OXPHOS dysfunction in widely different organisms (yeast, *Drosophila*, mammalian tissues and cell-lines) seems to be a remodeling of gene expression connected with metabolism. Surprisingly, many changes appear to be directed more at maintaining redox balance and the pool of carbon skeletons needed for biosynthesis, rather than at boosting ATP production. This contrasts with the more traditional view of OXPHOS as a machine for highly efficient ATP synthesis [106] and emphasizes the long-standing observation that glycolysis is a preferred pathway in highly proliferative cells even when oxygen is present [107]. Upregulation of the HIG1 homologue CG17734 in *tko^25t^* may further enhance this effect.

The overall response resembles one expected in cases of starvation. One way to mitigate the effects of pathological OXPHOS inhibition might thus be to provide nutritional supplements that alleviate the deficiency, in which case it is vital to determine exactly which metabolites are most affected. It may be, for example, that manipulating the balance between different types of nutrient might have more effect than changing their overall caloric amount. A second aspect is the use of alternate pathways for NADH re-oxidation which, in *tko^25t^*, as in mitochondrial disease patients, involves an upregulation of the shunt to lactate. The use of dichloroacetate (DCA), an activator of PDC, as a therapy for lactic acidosis [108] may thus be questioned, if the lactate shunt is essential for maintaining redox homeostasis. Whilst promoting a drop in circulating lactate levels, DCA does not appear to affect the major clinical features of disease [108]. Diverting pyruvate back into the TCA cycle will not alleviate the block on NADH reoxidation, and may even be harmful [80], if entraining increased mitochondrial ROS production.

Alternative strategies for reoxidizing NADH, such as the use of a metabolic by-pass [109], might be more fruitful. In plants, a major target of the retrograde response is the induction of the alternative oxidase (AOX) and the non-proton-pumping NADH dehydrogenases, which provide a substitute pathway to maintain redox balance and mitochondrial intermediary metabolism under conditions of OXPHOS inhibition. Such conditions occur naturally in plants, due to the fact that, in the light, photosynthesis maintains ATP at a high level, which limits mitochondrial OXPHOS by coupling. Expression of the by-pass genes unblocks NADH reoxidation and the operation of the TCA cycle to serve the needs of biosynthesis. Increased intramitochondrial ROS production has been postulated as a primary inducing signal of retrograde responses in plants [110].

A ketogenic diet has also been proposed to alleviate some symptoms of mitochondrial diseases [108], [109], especially where oxidative stress is a key pathological mechanism [112]. In our study we did not find evidence for chronic oxidative stress being a generalized consequence of OXPHOS dysfunction in *tko^25t^*. However, dietary supplementation by specific fatty acids and/or specific amino acids might bring benefits for the reasons already discussed. The elimination of carbohydrates from the diet might, conversely, have deleterious or even catastrophic consequences, since glucose as a fuel seems even more essential under conditions of OXPHOS limitation. We would suggest that supplementation and modulation, rather than complete elimination of glucose might be more effective.

### Experimental Approaches to Retrograde Signalling

In plants, the signalling molecules and pathways involved in retrograde regulation have been studied using an AOX-luciferase reporter gene in *Arabidopsis* cell culture [113]. Over 100 independent mutants were isolated, altered both in global and more stress-specific responses. Although retrograde regulation may be more complex in plants, given the presence of two semi-autonomous organelles, some of the underlying signalling machinery may be common to plants and animals. The identification of some specific target genes in the present study offers the prospect of using a similar reporter strategy, combined with the genetic resources of *Drosophila*, to identify both the cell-autonomous and hormonal signalling pathways of retrograde regulation in a metazoan, with obvious possible relevance to finding new drug targets and other therapies for mitochondrial disease. It will also be important to check that the widespread transcriptional regulation documented in the present study is reflected also at the protein level. Other target genes in the affected pathways, as well as components of the signaling machinery that brings about the adaptive changes, may turn out to be regulated translationally or post-translationally, rather than at the RNA level, to unravel this will require an extensive further analysis using proteomic tools.

## Materials and Methods

### *Drosophila* Stocks, Maintenance, and Crosses

Wild type and *tko^25t^* mutant flies in both Canton S and Oregon R backgrounds were maintained in standard oatmeal and molasses medium containing 1.5% (w/v) sucrose (Merck), 3% glucose (Sigma), 3.5% Instant Dry Baker's Yeast (European), 1.5% Maize flour (Oriola), 1% Wheat germ (Oriola), 1% Soya flour (Oriola), 1% agar (Oriola) and 3% Lyle's black treacle (Tate & Lyle, UK), to which was added 0.1% Nipagin M (Sigma) and 0.5% (v/v) propionic acid (JT Baker). Crosses were set up as indicated in Figure 1.

### RNA Extraction

Three independent groups of 30 *tko^25t^* virgin females and another 3 independent groups of 30 virgin wild-type females were collected and aged to 1 day prior to RNA extraction. In parallel, 3 independent groups of 40 *tko^25t^* males and another 3 of wild-type males were collected and aged to 1 day. Each group of flies was homogenized independently in 1 ml of Trizol (Invitrogen) and RNA was extracted with chloroform and precipitated with 2-propanol by centrifugation at 12000 *g_max_* for 10 min at 4°C. After washing with 75% EtOH, RNA was resuspended in DEPC-treated water and cleaned using the RNeasy® MinElute™ Cleanup kit (Qiagen). RNA concentration and purity were checked spectrophotometrically and RNA quality was verified electrophoretically. RNA was stored at −20°C in RNase-free water.

### Probe Synthesis and Hybridization

A cRNA probe was synthesized and fragmented from each RNA sample using Affymetrix® protocols and kits provided by Qiagen. Each cRNA probe was then hybridized to independent GeneChip® Drosophila Genome 2.0 Arrays (Affymetrix). Hybridization was performed overnight in the GeneChip® Hybridization Oven 640 at 45°C with shaking at 60 rpm; washes and staining of the probe were performed in the GeneChip® fluidics station using buffers and protocols provided by the manufacturer; and the final high-quality scan of the arrays was performed with the GeneChip® Scanner 3000. The full system was controlled by the GeneChip® Operating Software (GCOS), and both the equipment and the software were provided by Affymetrix®.

### Statistical Analysis

Data extraction, cell intensity calculation and computational analysis were performed using GeneChip® Operating Software and Expression Console Release Software from Affymetrix. For each pair-wise comparative analysis, the signal value (MAS5 and RMA algorithms, at linear scale) of each probe was compared as follows: wild-type to *tko^25t^* in the both sexes. The statistics was performed with Microsoft® Office Excel 2003. Firstly, the data were prefiltered according to their detection p-value, using those signal-probes with p-value<0.05. Secondly, SAM (Significance Analysis of Microarrays, Stanford Tools) was performed as described by Tusher *et al.* [114] using s0 = 20 and minimum full change (R) = 1.5 as fixed parameters, and Δ-value so that False Discovery Rate (FDR)<2.5%. Finally, regulated probes in both male and female *tko^25t^*–wild type analysis were selected and classified according to their gene ontology. The data have been deposited with the NCBI GEO database (Series record GSE10169).

### Quantitative RT-PCR Analysis of mRNA

RNA was reverse transcribed using random hexamers and M-Mul V Reverse Transcriptase (Fermentas). Real-time PCR was run in triplicate on a Light Cycler (Roche) using the SYBR Green PCR kit (Qiagen) and primers as detailed in File S1. Parallel reactions for *RpL32* (rp49) were used as a standardization control. The raw fluorescence data were extracted using Light Cycler data collection software version 3.5 (Roche) and analyzed according to manufacturer's instructions for baseline adjustment and noise reduction.

### Courtship and Egg-Laying Analyses

Males and virgin females were collected after eclosion, placed in separate vials (10 animals/vial) and stored over five days, with transfer to fresh food-containing vials every second day. For courtship analysis, single males and females were placed together in a food-containing vial, and the proportion of successful copulation was observed over 1 h. At least 15 females were observed per experiment. To assay oviposition, single mated females were transferred daily to fresh food-containing vials and the number of eggs laid by individual females was counted in each 24 h period. Between 15 and 20 mated females were included in each experiment. Each experiment was repeated three times.

### PCR Test for *Wolbachia* Infection

Total DNA was extracted from different *Drosophila* stocks (see Figure 2) as previously [18] and amplified with the following primer pairs and amplification conditions: Wolbachia 16S rDNA-F, 5′- TTGTAGCCTGCTATGGTATAACT-3′, Wolbachia 16S rDNA-R, 5′- GAATAGGTATGATTTTCATGT-3′, denaturation 95°C 1 min, annealing 52°C 1 min, extension 72°C 1 min, 35 cycles; mitochondrial 12S rDNA-F, 5′- TTTGGCGGTATTTTAGTAT - 3′, mitochondrial 12S rDNA-R, 5′- CTTAAATATAAGCTACACCTTGATC - 3′, denaturation 95°C 45 s, annealing 56°C 45 s, extension 72°C 1 min, 35 cycles, after which samples were mixed and analysed by 1% agarose gel electrophoresis.

## Supporting Information

File S1 Supplementary text including additional information on materials and methods.
(0.01 MB PDF)

Table S1 Gene ontology categories used to classify the selected genes according to their biological process, molecular function, and cellular component.
(0.03 MB XLS)

Table S2 Regulated genes in *tko^25t^* mutant flies.
(0.03 MB XLS)

Table S3 Changes in gene expression according to functional category.
(0.17 MB XLS)

Table S4 Fold-change (calculated as significance analysis of microarrays) of unselected genes.
(0.04 MB XLS)

Table S5 *tko^25t^*-regulated genes with possible roles in hormonal signalling of mitochondrial dysfunction.
(0.02 MB XLS)

Table S6 Changes in expression in *tko^25t^* of homologues of genes modulated in other oxidative phosphorylation disease models.
(0.14 MB XLS)

Figure S1 Congruence in patterns of changes in gene expression in *tko^25t^* flies. In each field are denoted the number of changes in the stated directions at the intermediate filtering stringency condition (see Tables 1 and 2). The relative numbers of changes in each category are shown in the boxes, and denoted by color intensity, from red to pale yellow. A higher proportion of down-regulated genes than up-regulated genes are altered sex-specifically, but many of these are already expressed in a sex-specific manner.
(0.04 MB PDF)

Figure S2 Aligned amino acid sequences of the Rieske iron-sulfur protein variants. The two *Drosophila melanogaster* variant proteins, RFeSP-PA (NCBI database accession number AAF51353, blue) and RFeSP-PB (NCBI database accession number AAF51354, red) are shown aligned with the sequence of *Saccharomyces cerevisiae* Rip1p (NCBI database accession number NP_010890, black), using the one-letter amino acid code. Identical amino acids are boxed in pale blue. RFeSP-PB is homologous with Rip1p throughout its length, whereas the carboxy-terminal one-third of RFe-SP-PA is unrelated. The point of divergence with RFeSP-PB is arrowed, and the Rieske domain (http://www.ncbi.nlm.nih.gov/Structure/cdd/cddsrv.cgi?uid=58540) is underlined.
(0.00 MB PDF)

Figure S3 Sex determination hierarchy in *Drosophila*.
(0.01 MB PDF)

## References

[1] Schon EA (2000). Mitochondrial genetics and disease.. Trends Biochem Sci.

[2] Smeitink JA (2003). Mitochondrial disorders: Clinical presentation and diagnostic dilemmas.. J Inher Metab Dis.

[3] Schon EA, Santra S, Pallotti F, Girvin ME (2001). Pathogenesis of primary defects in mitochondrial ATP synthesis.. Semi Cell Dev Biol.

[4] Triepels RH, Heuvel LPVD, Trijbels JM, Smeitink JA (2001). Respiratory chain complex I deficiency.. Am J Med Genet.

[5] Kirino Y, Suzuki T (2005). Human Mitochondrial Diseases Associated with tRNA Wobble Modification Deficiency.. RNA Biol.

[6] Copeland WC (2008). Inherited Mitochondrial Diseases of DNA Replication.. Annu Rev Med.

[7] Hakonen AH, Davidzon G, Salemi R, Bindoff LA, Van Goethem G (2007). Abundance of the POLG disease mutations in Europe, Australia, New Zealand, and the United States explained by single ancient European founders.. Eur J Hum Genet.

[8] Miller C, Saada A, Shaul N, Shabtai N, Ben-Shalom E (2004). Defective mitochondrial translation caused by a ribosomal protein (MRPS16) mutation.. Ann Neurol.

[9] Saada A, Shaag A, Arnon S, Dolfin T, Miller C (2007). Antenatal mitochondrial disease caused by mitochondrial ribosomal protein (MRPS22) mutation.. J Med Genet.

[10] Shoubridge EA (2001). Cytochrome c oxidase deficiency.. Am J Med Genet.

[11] O'Brien TW (2003). Properties of human mitochondrial ribosomes.. IUBMB Life.

[12] Smeitink J, van den Heuvel L, DiMauro S (2001). The genetics and pathology of oxidative phosphorylation.. Nat Rev Genet.

[13] Shah ZH, O'Dell KM, Miller SC, An X, Jacobs HT (1997). Metazoan nuclear genes for mitoribosomal protein S12.. Gene.

[14] Toivonen JM, Boocock MR, Jacobs HT (1999). Modelling in Escherichia coli of mutations in mitoribosomal protein S12: novel mutant phenotypes of rpsL.. Mol Microbiol.

[15] Royden CS, Pirrotta V, Jan LY (1987). The tko locus, site of a behavioral mutation in D. melanogaster, codes for a protein homologous to prokaryotic ribosomal protein S12.. Cell.

[16] Zhang YQ, Roote J, Brogna S, Davis AW, Barbash DA (1999). stress sensitive B encodes an adenine nucleotide translocase in Drosophila melanogaster.. Genetics.

[17] Fergestad T, Bostwick B, Ganetzky B (2006). Metabolic disruption in Drosophila bang-sensitive seizure mutants.. Genetics.

[18] Toivonen JM, O'Dell KM, Petit N, Irvine SC, Knight GK (2001). Technical knockout, a Drosophila model of mitochondrial deafness.. Genetics.

[19] Toivonen JM, Manjiry S, Touraille S, Alziari S, O'Dell KMC (2003). Gene dosage and selective expression modify phenotype in a Drosophila model of human mitochondrial disease.. Mitochondrion.

[20] Kemppainen E, Fernández-Ayala DJ, Galbraith LC, O'Dell KM, Jacobs HT (2009). Phenotypic suppression of the Drosophila mitochondrial disease-like mutant *tko^25t^* by duplication of the mutant gene in its natural chromosomal context.. Mitochondrion.

[21] Traven A, Wong JMS, Xu D, Sopta M, Ingles CJ (2001). Interorganelle communication. Altered nuclear gene expression profiles in a yeast mitochondrial DNA mutant.. J Biol Chem.

[22] Liu Z, Butow RA (2006). Mitochondrial Retrograde Signaling.. Annu Rev of Genet.

[23] Sardiello M, Licciulli F, Catalano D, Attimonelli M, Caggese C (2003). MitoDrome: a database of Drosophila melanogaster nuclear genes encoding proteins targeted to the mitochondrion.. Nucl Acids Res.

[24] Spradling AC, Stern D, Beaton A, Rehm EJ, Laverty T (1999). The Berkeley Drosophila Genome Project Gene Disruption Project: Single P-Element Insertions Mutating 25% of Vital Drosophila Genes.. Genetics.

[25] Ross J, Jiang H, Kanost MR, Wang Y (2003). Serine proteases and their homologs in the Drosophila melanogaster genome: an initial analysis of sequence conservation and phylogenetic relationships.. Gene.

[26] Wang S, Magoulas C, Hickey D (1999). Concerted evolution within a trypsin gene cluster in Drosophila.. Mol Biol Evol.

[27] Carlson JR, Hogness DS (1985). The Jonah genes: A new multigene family in Drosophila melanogaster.. Dev Biol.

[28] Didelot G, Molinari F, Tchenio P, Comas D, Milhiet E (2006). Tequila, a Neurotrypsin Ortholog, Regulates Long-Term Memory Formation in *Drosophila*.. Science.

[29] Molinari F, Rio M, Meskenaite V, Encha-Razavi F, Auge J (2002). Truncating Neurotrypsin Mutation in Autosomal Recessive Nonsyndromic Mental Retard.. Sci.

[30] Verheijen FW, Verbeek E, Aula N, Beerens CEMT, Havelaar AC (1999). A new gene, encoding an anion transporter, is mutated in sialic acid storage diseases.. Nat Genet.

[31] Nezu JI, Tamai I, Oku A, Ohashi R, Yabuuchi H (1999). Primary systemic carnitine deficiency is caused by mutations in a gene encoding sodium ion-dependent carnitine transporter.. Nat Genet.

[32] Tamai I, Yabuuchi H, Nezu JI, Sai Y, Oku A (1997). Cloning and characterization of a novel human pH-dependent organic cation transporter, OCTN1.. FEBS Lett.

[33] Pletcher SD, Macdonald SJ, Marguerie R, Certa U, Stearns SC (2002). Genome-Wide Transcript Profiles in Aging and Calorically Restricted Drosophila melanogaster.. Curr Biol.

[34] Zinke I, Schutz CS, Katzenberger JD, Bauer M, Pankratz MJ (2002). Nutrient control of gene expression in Drosophila: microarray analysis of starvation and sugar-dependent response.. EMBO J.

[35] Kurapati R, Passananti HB, Rose MR, Tower J (2000). Increased hsp22 RNA Levels in Drosophila Lines Genetically Selected for Increased Longevity.. J Gerontol Biol Sci Med Sci.

[36] Morrow G, Battistini S, Zhang P, Tanguay RM (2004). Decreased Lifespan in the Absence of Expression of the Mitochondrial Small Heat Shock Protein Hsp22 in Drosophila.. J Biol Chem.

[37] Morrow G, Samson M, Michaud S, Tanguay RM (2004). Overexpression of the small mitochondrial Hsp22 extends Drosophila life span and increases resistance to oxidative stress.. FASEB J.

[38] Landis GN, Abdueva D, Skvortsov D, Yang J, Rabin BE (2004). Similar gene expression patterns characterize aging and oxidative stress in Drosophila melanogaster.. Proc Natl Acad Sci U S A.

[39] Tiranti V, D'Adamo P, Briem E, Ferrari G, Mineri R (2004). Ethylmalonic encephalopathy is caused by mutations in ETHE1, a gene encoding a mitochondrial matrix protein.. Am J Hum Genet.

[40] Tiranti V, Viscomi C, Hildebrandt T, Di Meo I, Mineri R (2009). Loss of ETHE1, a mitochondrial dioxygenase, causes fatal sulfide toxicity in ethylmalonic encephalopathy.. Nat Med.

[41] Thornalley PJ (1993). The Glyoxalase System in Health and Disease.. Mol Asp Med.

[42] Bandi C, Dunn AM, Hurst GDD, Rigaud T (2001). Inherited microorganisms, sex-specific virulence and reproductive parasitism.. Trends Parasitol.

[43] Tzou P, Reichhart JM, Lemaitre B (2002). Constitutive expression of a single antimicrobial peptide can restore wild-type resistance to infection in immunodeficient Drosophila mutants.. Proc Natl Acad Sci U S A.

[44] Uttenweiler-Joseph S, Moniatte M, Lagueux M, Van Dorsselaer A, Hoffmann JA (1998). Differential display of peptides induced during the immune response of Drosophila: A matrix-assisted laser desorption ionization time-of-flight mass spectrometry study.. Proc Natl Acad Sci U S A.

[45] Levashina EA, Ohresser S, Bulet P, Reichhart JM, Hetru C (1995). Metchnikowin, a Novel Immune-Inducible Proline-Rich Peptide from Drosophila with Antibacterial and Antifungal Properties.. Eur J Biochem.

[46] Clark AG, Doane WW (1983). Desiccation Tolerance of the Adipose60 Mutant of Drosophila-Melanogaster.. Hereditas.

[47] Goto SG (2001). A novel gene that is up-regulated during recovery from cold shock in Drosophila melanogaster.. Gene.

[48] Häder T, Muller S, Aguilera M, Eulenberg KG, Steuernagel A (2003). Control of triglyceride storage by a WD40/TPR-domain protein.. EMBO Rep.

[49] Teague BD, Clark AG, Doane WW (1986). Developmental Analysis of Lipids from Wild-Type and *Adipose60* Mutants of *Drosophila-Melanogaste*r.. J Exp Zool.

[50] Buchon N, Broderick NA, Poidevin M, Pradervand S, Lemaitre B (2009). Drosophila intestinal response to bacterial infection: activation of host defense and stem cell proliferation.. Cell Host Microbe.

[51] Wang J, Cao Y, Chen Y, Chen YM, Gardner P (2006). Pancreatic beta cells lack a low glucose and O-2-inducible mitochondrial protein that augments cell survival.. Proc Natl Acad Sci U S A.

[52] Pouliot JJ, Yao KC, Robertson CA, Nash HA (1999). Yeast gene for a Tyr-DNA phosphodiesterase that repairs topoisomerase I complexes.. Science.

[53] Nitiss KC, Malik M, He XP, White SW, Nitiss JL (2006). Tyrosyl-DNA phosphodiesterase (Tdp1) participates in the repair of Top2-mediated DNA damage.. Proc Natl Acad Sci U S A.

[54] Dunlop J, Morin X, Corominas M, Serras F, Tear G (2004). glaikit is essential for the formation of epithelial polarity and neuronal development.. Curr Biol.

[55] Yasui A, Eker APM, Yasuhira S, Yajima H, Kobayashi T (1994). A New Class of DNA Photolyases Present in Various Organisms Including Aplacental Mammals.. EMBO J.

[56] Benes H, Edmondson RG, Fink P, Kejzlarovalepesant J, Lepesant JA (1990). Adult Expression of the Drosophila Lsp-2 Gene.. Dev Biol.

[57] Deutsch J, Laval M, Lepesant JA, Maschat F, Pourrain F (1989). Larval Fat Body-Specific Gene-Expression in Drosophila-Melanogaster.. Developmental Genetics.

[58] Burmester T, Antoniewski C, Lepesant JA (1999). Ecdysone-regulation of synthesis and processing of Fat Body Protein 1, the larval serum protein receptor of Drosophila melanogaster.. Eur J Biochem.

[59] Karlsson C, Korayem AM, Scherfer C, Loseva O, Dushay MS (2004). Proteomic analysis of the Drosophila larval hemolymph clot.. J Biol Chem.

[60] Arbeitman MN, Furlong EEM, Imam F, Johnson E, Null BH (2002). Gene expression during the life cycle of Drosophila melanogaster.. Science.

[61] Gagou ME, Kapsetaki M, Turberg A, Kafetzopoulos D (2002). Stage-specific expression of the chitin synthase DmeChSA and DmeChSB genes during the onset of Drosophila metamorphosis.. Insect Biochem Mol Biol.

[62] Boya P, Gonzalez-Polo RA, Casares N, Perfettini JL, Dessen P (2005). Inhibition of macroautophagy triggers apoptosis.. Mol Cell Biology.

[63] Lum JJ, Bauer DE, Kong M, Harris MH, Li C (2005). Growth factor regulation of autophagy and cell survival in the absence of apoptosis.. Cell.

[64] Szakmary A, Cox DN, Wang Z, Lin HF (2005). Regulatory relationship among piwi, pumilio, and bag-of-marbles in Drosophila germline stem cell self-renewal and differentiation.. Curr Biol.

[65] Sarov-Blat L, So WV, Liu L, Rosbash M (2000). The Drosophila takeout gene is a novel molecular link between circadian rhythms and feeding behavior.. Cell.

[66] Lazareva AA, Roman G, Mattox W, Hardin PE, Dauwalder B (2007). A role for the adult fat body in Drosophila male courtship behavior.. PLoS Genet.

[67] Meunier N, Belgacem YH, Martin JR (2007). Regulation of feeding behaviour and locomotor activity by takeout in Drosophila.. J Exp Biol.

[68] Dauwalder B, Tsujimoto S, Moss J, Mattox W (2002). The Drosophila takeout gene is regulated by the somatic sex-determination pathway and affects male courtship behavior.. Genes Dev.

[69] Lorenz LJ, Hall JC, Rosbash M (1989). Expression of a Drosophila Messenger-Rna Is under Circadian Clock Control during Pupation.. Development.

[70] Fujii S, Amrein H (2002). Genes expressed in the Drosophila head reveal a role for fat cells in sex-specific physiology.. EMBO J.

[71] Wolfner MF (2003). Sex determination: Sex on the brain?. Curr Biol.

[72] Hou XS, Perrimon N (1997). The JAK-STAT pathway in Drosophila.. Trends Genet.

[73] Kandel ES, Hay N (1999). The regulation and activities of the multifunctional serine/threonine kinase Akt/PKB.. Exp Cell Res.

[74] Scanga SE, Ruel L, Binari RC, Snow B, Stambolic V (2000). The conserved PI3 ' K/PTEN/Akt signaling pathway regulates both cell size and survival in Drosophila.. Oncogene.

[75] Ruel L, Pantesco V, Lutz Y, Simpson P, Bourouis M (1993). Functional-Significance of a Family of Protein-Kinases Encoded at the Shaggy Locus in Drosophila.. EMBO J.

[76] Kanuka H, Kuranaga E, Takemoto K, Hiratou T, Okano H (2005). Drosophila caspase transduces Shaggy/GSK-3 beta kinase activity in neural precursor development.. EMBO J.

[77] Martinek S, Inonog S, Manoukian AS, Young MW (2001). A role for the segment polarity gene shaggy/GSK-3 in the Drosophila circadian clock.. Cell.

[78] Wolf FW, Eddison M, Lee S, Cho W, Heberlein U (2007). GSK-3/Shaggy regulates olfactory habituation in Drosophila.. Proc Natl Acad Sci U S A.

[79] Mackay TFC, Heinsohn SL, Lyman RF, Moehring AJ, Morgan TJ (2005). Genetics and genomics of Drosophila mating behavior.. Proc Natl Acad Sci U S A.

[80] Kaufmann P, Engelstad K, Wei Y, Jhung S, Sano MC (2006). Dichloroacetate causes toxic neuropathy in MELAS - A randomized, controlled clinical trial.. Neurology.

[81] Cross DAE, Alessi DR, Cohen P, Andjelkovich M, Hemmings BA (1995). Inhibition of Glycogen-Synthase Kinase-3 by Insulin-Mediated by Protein-Kinase-B.. Nature.

[82] Shaw M, Cohen P, Alessi DR (1997). Further evidence that the inhibition of glycogen synthase kinase-3 beta by IGF-1 is mediated by PDK1/PKB-induced phosphorylation of Ser-9 and not by dephosphorylation of Tyr-216.. FEBS Lett.

[83] Bennett D, Szoor B, Gross S, Vereshchagina N, Alphey L (2003). Ectopic expression of inhibitors of protein phosphatase type 1 (PP1) can be used to analyze roles of PP1 in Drosophila development.. Genetics.

[84] Todres E, Nardi JB, Robertson HM (2000). The tetraspanin superfamily in insects.. Insect Mol Biol.

[85] Stacpoole PW, Henderson GN, Yan ZM, Cornett R, James MO (1998). Pharmacokinetics, metabolism, and toxicology of dichloroacetate.. Drug Metab Rev.

[86] De Virgilio C, Loewith R (2006). Cell growth control: little eukaryotes make big contributions.. Oncogene.

[87] Lin JD, Handschin C, Spiegelman BM (2005). Metabolic control through the PGC-1 family of transcription coactivators.. Cell Metab.

[88] Lochhead PA, Salt IP, Walker KS, Hardie DG, Sutherland C (2000). 5-aminoimidazole-4-carboxamide riboside mimics the effects of insulin on the expression of the 2 key gluconeogenic genes PEPCK and glucose-6-phosphatase.. Diabetes.

[89] Rencurel F, Stenhouse A, Hawley SA, Friedberg T, Hardie DG (2005). AMP-activated protein kinase mediates phenobarbital induction of CYP2B gene expression in hepatocytes and a newly derived human hepatoma cell line.. J Biol Chem.

[90] Rencurel F, Foretz M, Kaufmann MR, Stroka D, Looser R (2006). Stimulation of AMP-activated protein kinase is essential for the induction of drug metabolizing enzymes by phenobarbital in human and mouse liver.. Mol Pharmacol.

[91] Wang J, Cao Y, Chen Y, Chen Y, Gardner P (2006). Pancreatic beta cells lack a low glucose and O2-inducible mitochondrial protein that augments cell survival.. Proc Natl Acad Sci U S A.

[92] Bischoff V, Vignal C, Boneca IG, Michel T, Hoffmann JA (2004). Function of the drosophila pattern-recognition receptor PGRP-SD in the detection of Gram-positive bacteria.. Nat Immunol.

[93] De Gregorio E, Spellman PT, Rubin GM, Lemaitre B (2001). Genome-wide analysis of the Drosophila immune response by using oligonucleotide microarrays.. Proc Natl Acad Sci U S A.

[94] Ekengren S, Hultmark D (2001). A family of Turandot-related genes in the humoral stress response of Drosophila.. Biochem Biophys Res Commun.

[95] Graham LA, Davies PL (2002). The odorant-binding proteins of Drosophila melanogaster: annotation and characterization of a divergent gene family.. Gene.

[96] Kurucz E, Markus R, Zsamboki J, Folkl-Medzihradszky K, Darula Z (2007). Nimrod, a putative phagocytosis receptor with EGF repeats in *Drosophila* plasmatocytes.. Curr Biol.

[97] van Waveren C, Sun YB, Cheung HS, Moraes CT (2006). Oxidative phosphorylation dysfunction modulates expression of extracellular matrix - remodeling genes and invasion.. Carcinogenesis.

[98] Alemi M, Prigione A, Wong A, Schoenfeld R, DiMauro S (2007). Mitochondrial DNA deletions inhibit proteasomal activity and stimulate an autophagic transcript.. Free Rad Biol Med.

[99] Roof DJ, Hayes A, Adamian M, Chishti AH, Li TS (1997). Molecular characterization of abLIM, a novel actin-binding and double zinc finger protein.. J Cell Biol.

[100] Okabe M, Okano H (1997). Two-step induction of chordotonal organ precursors in Drosophila embryogenesis.. Development.

[101] Boltz KA, Ellis LL, Carney GE (2007). *Drosophila melanogaster* p24 genes have developmental, tissue-specific, and sex-specific expression patterns and functions.. Dev Dyn.

[102] Hansson A, Hance N, Dufour E, Rantanen A, Hultenby K (2004). A switch in metabolism precedes increased mitochondrial biogenesis in respiratory chain-deficient mouse hearts.. Proc Natl Acad Sci U S A.

[103] Prigione A, Cortopassi G (2007). Mitochondrial DNA deletions induce the adenosine monophosphate-activated protein kinase energy stress pathway and result in decreased secretion of some proteins.. Aging Cell.

[104] Molina MR, Cripps RM (2001). Ostia, the inflow tracts of the Drosophila heart, develop from a genetically distinct subset of cardial cells.. Mech Dev.

[105] Tao Y, Schulz RA (2007). Heart development in Drosophila.. Sem Cell Dev Biol.

[106] Lehninger AL, Nelson DL, Cox MM (2004). Principles of Biochemistry, Fourth Edition..

[107] Warburg O (1956). Origin of Cancer Cells.. Science.

[108] Stacpoole PW, Kerr DS, Barnes C, Bunch ST, Carney PR (2006). Controlled clinical trial of dichloroacetate for treatment of congenital lactic acidosis in children.. Pediatrics.

[109] Yagi T, Seo BB, Nakamaru-Ogiso E, Marella M, Barber-Singh J (2006). Possibility of transkingdom gene therapy for Complex I diseases.. Biochim Biophys Acta′.

[110] Rhoads DM, Subbaiah CC (2007). Mitochondrial retrograde regulation in plants.. Mitochondrion.

[111] Santra S, Gilkerson RW, Davidson M, Schon EA (2004). Ketogenic treatment reduces deleted mitochondrial DNAs in cultured human cells.. Ann Neurol.

[112] Zhao Z, Lange DJ, Voustianiouk A, MacGrogan D, Ho L (2006). A ketogenic diet as a potential novel therapeutic intervention in amyotrophic lateral sclerosis.. BMC Neurosci.

[113] Zarkovic J, Anderson SL, Rhoads DM (2005). A reporter gene system used to study developmental expression of alternative oxidase and isolate mitochondrial retrograde regulation mutants in Arabidopsis.. Plant Mol Biol.

[114] Tusher VG, Tibshirani R, Chu G (2001). Significance analysis of microarrays applied to the ionizing radiation response.. Proc Natl Acad Sci U S A.

[115] Jacobs HT, Fernandez-Ayala DJM, Manjiry S, Kemppainen E, Toivonen JM (2004). Mitochondrial disease in flies.. Biochim Biophys Acta.

