# Supplementary material for: Gene Expression in a Drosophila Model of Mitochondrial Disease
Source: PLoS One. 2010 Jan 6;5(1):e8549. doi: 10.1371/journal.pone.0008549 (PMC2798955; doi:10.1371/journal.pone.0008549)
Supplement: File S1 — Supplementary text including additional information on materials and methods. (0.01 MB PDF) [file pone.0008549.s001.pdf]

Gene expression in a *Drosophila* model of mitochondrial disease

Daniel J M Fernández-Ayala, Shanjun Chen, Esko Kemppainen, Kevin M C O'Dell

& Howard T Jacobs

SUPPLEMENTARY FILE S1

SUPPLEMENT TO MATERIALS AND METHODS

Primer pairs used for Q-RT-PCR

For standardization *RpL32* primers were used: forward, agcatacaggcccaagatcgtgaagaa; reverse, cacgttgtgcaccaggaacttcttgaa (both 5´ to 3´). Primers for the test genes were as follows (all 5´to 3´):

**Gene/transcript**	**Flybase ID**	**Forward primer**	**Reverse primer**
takeout	FBtr0084810	gtcccgtgggcataactcta	ttgccctggatgttataggg
Hsp22	FBtr0100558	tggctatagctccaggcact	agtctgctcgatggtcacct
Tequila-RA, -RB, -RD	FBtr0076526	gatattgccctggtggtgtt	cgacatggctgatccataca
CG11659	FBtr0083797	ggagagataattttccacgagatg	gccttgtgaccttgtagatctttt
Fbp1	FBtr0075749	tgggtttggtgagaggagac	ctggatgtcctggttgtcct
Obp99b	FBtr0085462	aaggttctcatcgttctcctattg	gggtactgccacttcttgtacttc
ImpL3	FBtr0077008	actttctgaagaacccccagat	taagcaaaatggtatcgggact
Pepck	FBtr0086701	gacgacattgcgtggatgaa	catggcaatgggattggtct

ADDITIONAL DETAILS OF REGULATED GENES

Note that, in some cases (see Supplementary Tables 3), we moved genes to a different functional category than the one implied by current genomic annotation, because of what we consider to be errors in this annotation. In other cases, where assignments are more debatable, we have retained current classification.
