# Supplementary figures and images for: Gene Expression in a Drosophila Model of Mitochondrial Disease
Source: PLoS One. 2010 Jan 6;5(1):e8549. doi: 10.1371/journal.pone.0008549 (PMC2798955; doi:10.1371/journal.pone.0008549)

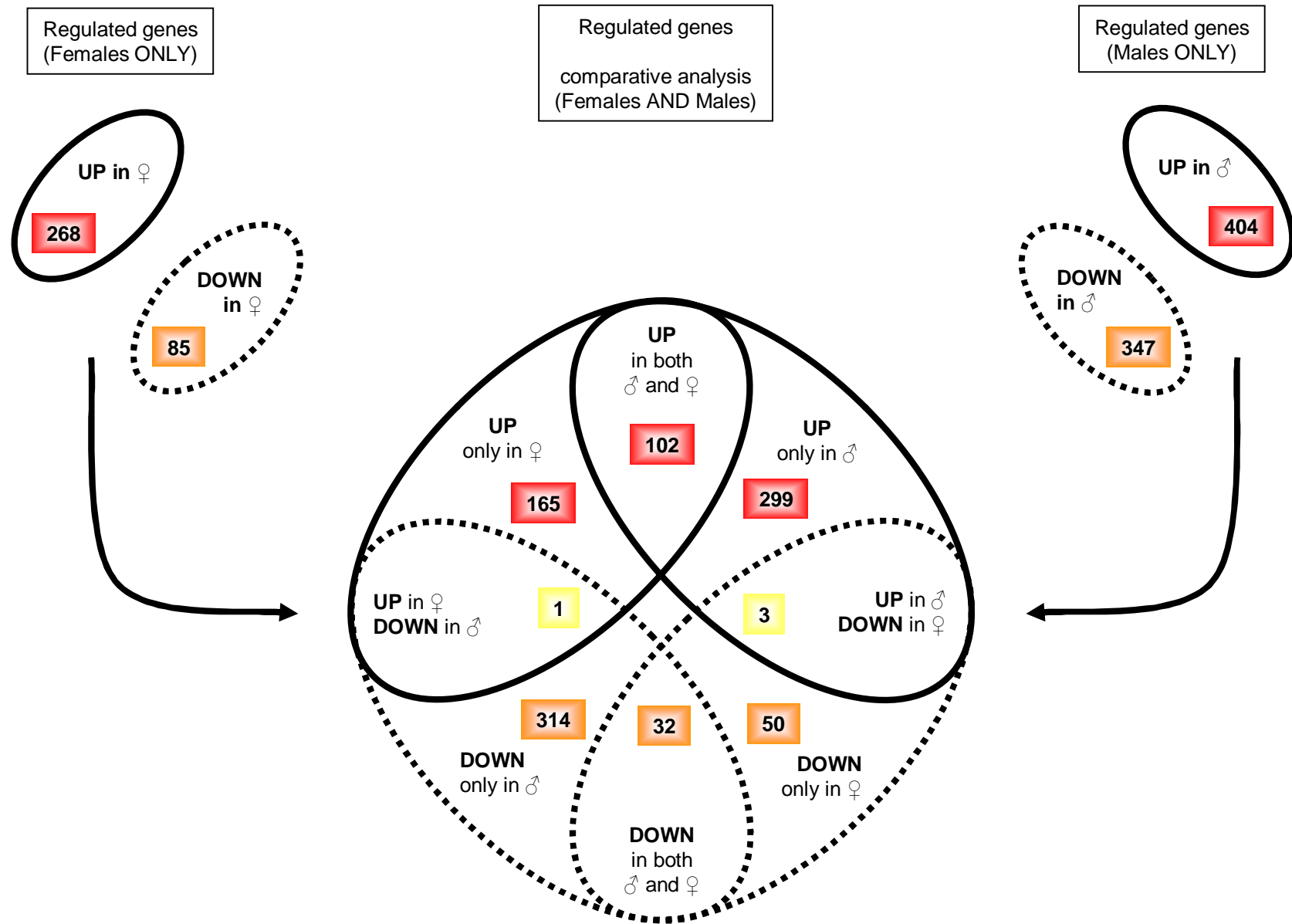

Figure S1, Fernández-Ayala *et al*

Supplement: Figure S1 — Congruence in patterns of changes in gene expression in tko25t flies. In each field are denoted the number of changes in the stated directions at the intermediate filtering stringency condition (see Tables 1 and 2). The relative numbers of changes in each category are shown in the boxes, and denoted by color intensity, from red to pale yellow. A higher proportion of down-regulated genes than up-regulated genes are altered sex-specifically, but many of these are already expressed in a sex-specific manner. (0.04 MB PDF) [file pone.0008549.s008.pdf]

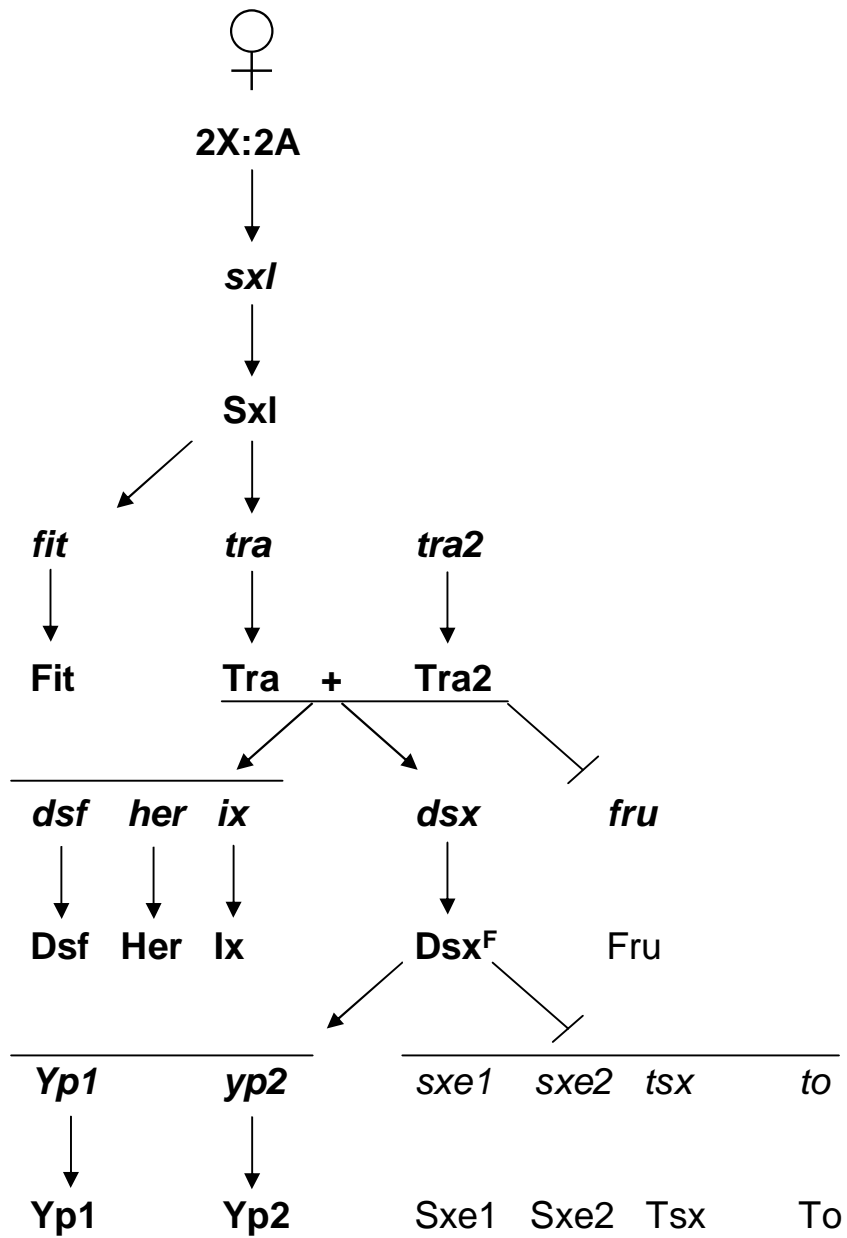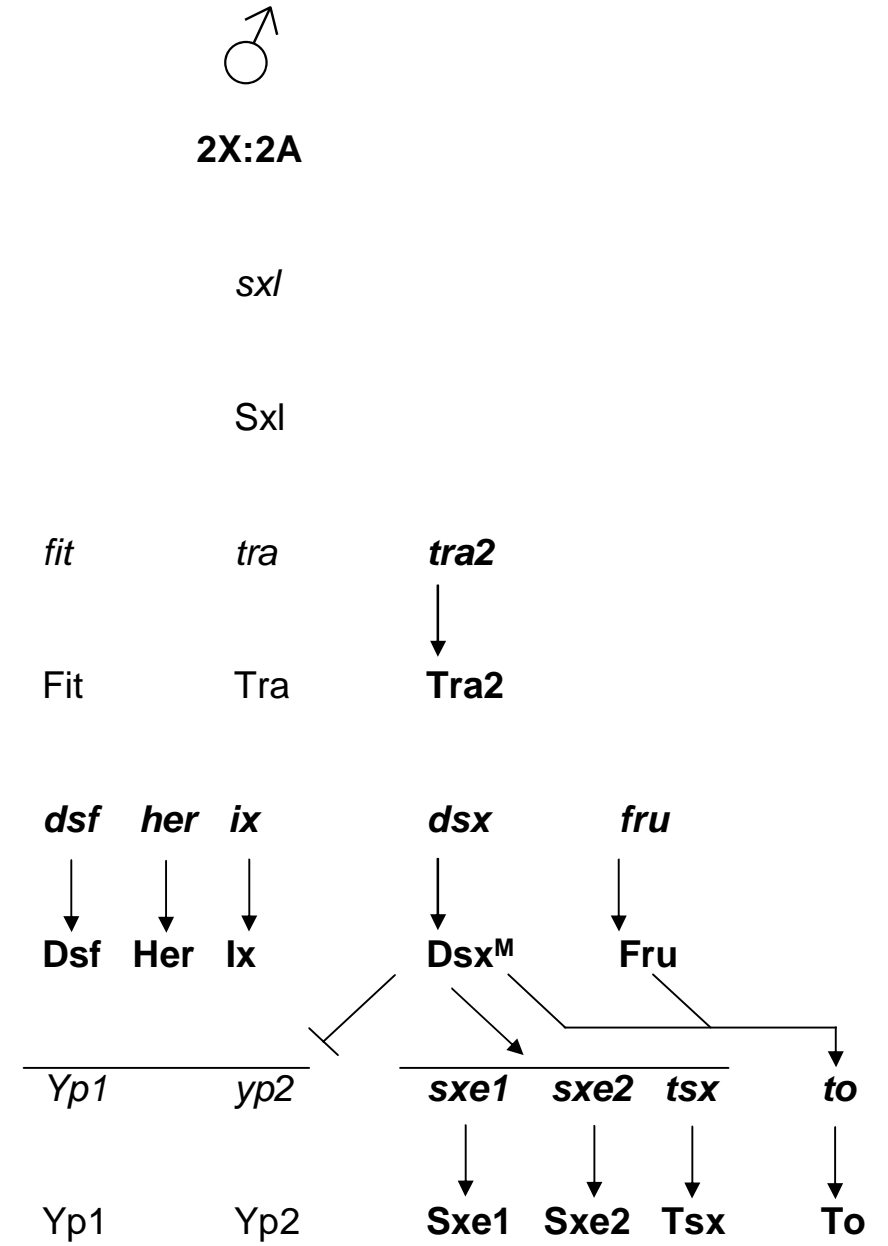

Supplement: Figure S3 — Sex determination hierarchy in Drosophila. (0.01 MB PDF) [file pone.0008549.s010.pdf]
