# Supplementary material for: Gene Expression in a Drosophila Model of Mitochondrial Disease
Source: PLoS One. 2010 Jan 6;5(1):e8549. doi: 10.1371/journal.pone.0008549 (PMC2798955; doi:10.1371/journal.pone.0008549)
Supplement: Figure S2 — Aligned amino acid sequences of the Rieske iron-sulfur protein variants. The two Drosophila melanogaster variant proteins, RFeSP-PA (NCBI database accession number AAF51353, blue) and RFeSP-PB (NCBI database accession number AAF51354, red) are shown aligned with the sequence of Saccharomyces cerevisiae Rip1p (NCBI database accession number NP_010890, black), using the one-letter amino acid code. Identical amino acids are boxed in pale blue. RFeSP-PB is homologous with Rip1p throughout its length, whereas the carboxy-terminal one-third of RFe-SP-PA is unrelated. The point of divergence with RFeSP-PB is arrowed, and the Rieske domain (http://www.ncbi.nlm.nih.gov/Structure/cdd/cddsrv.cgi?uid=58540) is underlined. (0.00 MB PDF) [file pone.0008549.s009.pdf]

```
RFeSP-PA     mmnavsrayvrggaqvlstglkasgvavnsmanrqahtdlqvpdfsayrresvkdsrrrn
RFeSP-PB     mmnavsrayvrggaqvlstglkasgvavnsmanrqahtdlqvpdfsayrresvkdsrrrn
Yeast Rip1p                mlgirssvktcfkpmsltskrlisqsllaskstyrtpnfddvlken

RFeSP-PA     dtaeerkafsylmvgagavggayaakglvntfigsmsasaevlamakieiklsdipegks
RFeSP-PB     dtaeerkafsylmvgagavggayaakglvntfigsmsasaevlamakieiklsdipegks
Yeast Rip1p  ndadkgrsyayfmvgamgllssagakstvetfissmtatadvlamakvevnlaaiplgkn

RFeSP-PA     vtfkwrgkplfirhrtaaeieternvptstlrdpeaddvgtvewvdslmllpisslatcd
RFeSP-PB     vtfkwrgkplfirhrtaaeieternvptstlrdpeaddqrvikpewlvvigvcthlgcvp
Yeast Rip1p  vvvkwqgkpvfirhrtpheiqeansvdmsalkdpqtdadrvkdpqwlimlgicthlgcvp

RFeSP-PA     qarvagghrsvhasglcahrerrrlgwlllplprlplrrlrkdpqgtrapqlggahprvp
RFeSP-PB     ianagdwggyycpchgshydasgrirkgpaplnlevpthefpnegllvvg
Yeast Rip1p  igeagdfggwfcpchgshydisgrirkgpaplnleipayefdgdkvivg

RFeSP-PA     qrgssrgrleellsfkppta
```

Figure S2, Fernández-Ayala *et al*
